# An Evo-Devo perspective on ever-growing teeth in mammals and dental stem cell maintenance

**DOI:** 10.3389/fphys.2014.00324

**Published:** 2014-08-28

**Authors:** Elodie Renvoisé, Frederic Michon

**Affiliations:** Developmental Biology Program, Institute of Biotechnology, University of HelsinkiHelsinki, Finland

**Keywords:** ever-growing tooth, stem cells, heterochrony, environment, tooth bioengineering

## Abstract

A major challenge for current evolutionary and developmental biology research is to understand the evolution of morphogenesis and the mechanisms involved. Teeth are well suited for the investigation of developmental processes. In addition, since teeth are composed of hard-mineralized tissues, primarily apatite, that are readily preserved, the evolution of mammals is well documented through their teeth in the fossil record. Hypsodonty, high crowned teeth with shallow roots, and hypselodonty, ever-growing teeth, are convergent innovations that have appeared multiple times since the mammalian radiation 65 million years ago, in all tooth categories (incisors, canines, premolars, and molars). A shift to hypsodonty, or hypselodonty, during mammalian evolution is often, but not necessarily, associated with increasingly abrasive diet during important environmental change events. Although the evolution of hypsodonty and hypselodonty is considered to be the result of heterochrony of development, little has been known about the exact developmental mechanisms at the origin of these morphological traits. Developmental biologists have been intrigued by the mechanism of hypselodonty since it requires the maintenance of continuous crown formation during development via stem cell niche activity. Understanding this mechanism may allow bioengineered tooth formation in humans. Hypsodonty and hypselodonty are thus examples of phenotypic features of teeth that have both impacts in understanding the evolution of mammals and holds promise for human tooth bioengineering.

## Introduction

Evo-Devo, or Evolutionary Developmental Biology, combines the two independent research disciplines of Evolutionary Biology and Developmental Biology (Arthur, 2002; Churchill, 2007; Gerson, 2007). Evolutionary Biology explores the evolution of forms that have been realized and their variability and Developmental Biology proposes morphogenetic mechanisms that could have been explored. Müller (2007b) insisted on the emergence of Evo-Devo from the limitations of these two disciplines to explain the form and the structure of the organisms. Since then, the Evo-Devo field became one of the most vigorous parts of biology (Gerson, 2007). In the recent years, considerable progress has been made in understanding the developmental basis of morphological evolution (Wagner, 2007). However, to become an independent scientific field, Evo-Devo must prove its potential to induce new scientific questions (Müller, 2007a). Among the new questions that can be assessed by Evo-Devo, one is how development contributes to phenotypic novelty (Müller, 2007a), and especially evolutionarily convergent phenotypes (Wake et al., 2011).

Mammalian dentition is characterized by heterodonty with four tooth categories (incisor, canine, premolar, and molar) and a great diversity of tooth morphology. These innovations in tooth morphology are considered to be in part responsible for the evolutionary success of mammals (Jernvall and Thesleff, 2012). While mammal ancestors, like vertebrates, were constantly replacing their teeth, and therefore qualified as polyphyodonts, most current mammals are diphyodont (replacing their teeth only once). At the beginning of the mammalian radiation, the stem mammal ancestors (e.g., *Morganucodon* and *Sinoconodon*) had five incisors, one canine, four premolars and five molars in each jaw quadrant but the number of functional incisors was variable, the canines could be replaced four time during the life-span of the animal, and the *Sinoconodon* molars were replaced, but not *Morganucodon* (Kielan-Jaworowska et al., 2004). Teeth were subsequently lost in almost all the placental mammalian lineages, such as humans and rodents (O'Leary et al., 2013), and teeth were replaced only once, except for molars that are never replaced. However, some exceptions can be found: one wallaby (*Petrogale concinna*), manatees and one African mole-rat (*Heliophobius argenteocinereus*) display a kind of polyphyodonty. These animals constantly replace their molars by developing new teeth in the back of their jaw, while the oldest molar is discarded in the front of the molar domain, resulting in a horizontal treadmill-like tooth replacement mechanism (Gomes Rodrigues et al., 2011).

**Figure 1:**
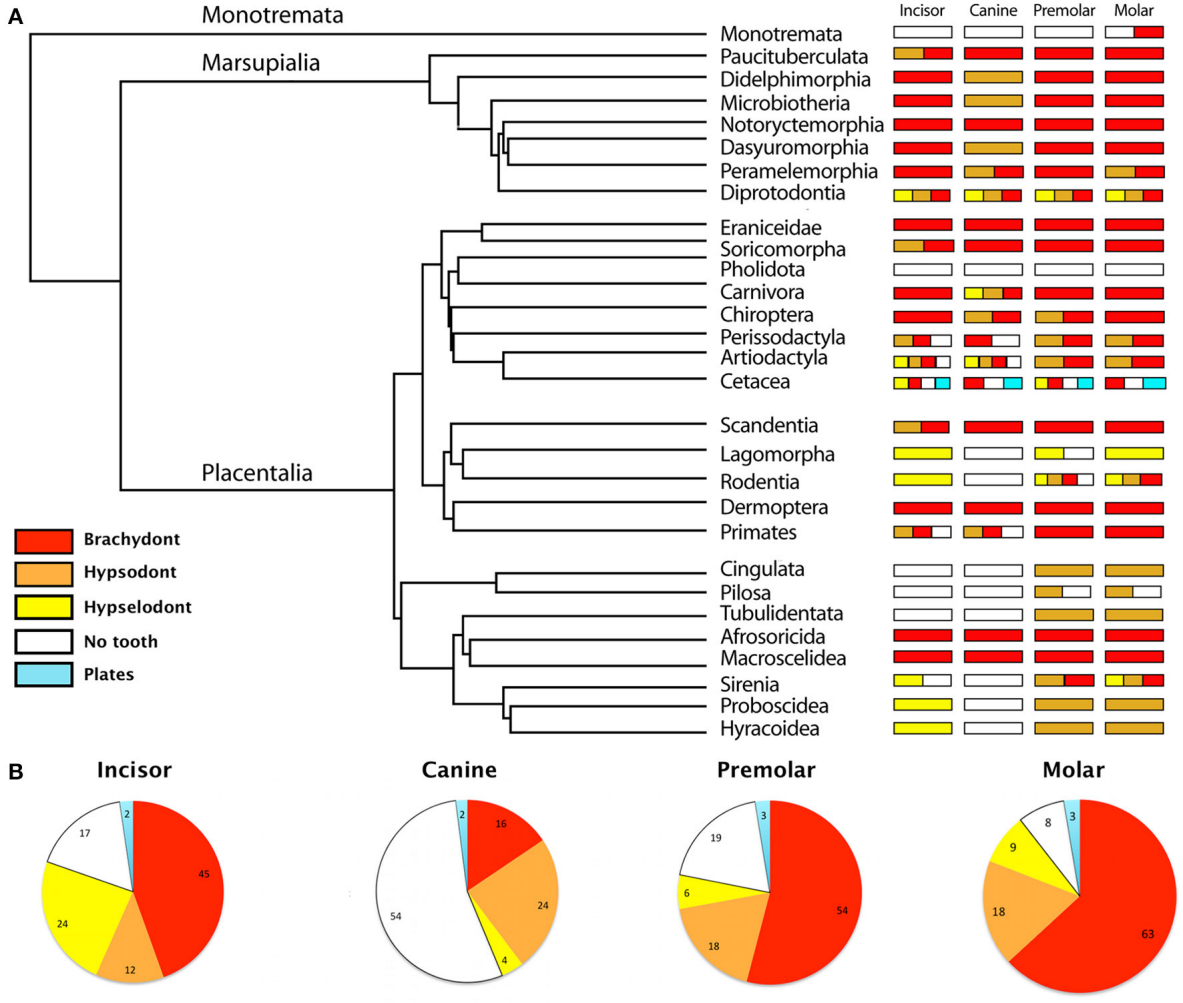
**Diversity of the tooth types—brachydont, hypsodont, hypselodont, no teeth and plates—in all mammalian Orders and Families**. **(A)** Phylogeny at the Order level, simplified from Meredith et al. (2011), associated with a schematic representation of the diversity of the tooth types in each mammalian Order. **(B)** Proportions of the tooth types in all mammalian Families. Results are represented in percent. The data refers to the Table S1.

**Figure 2:**
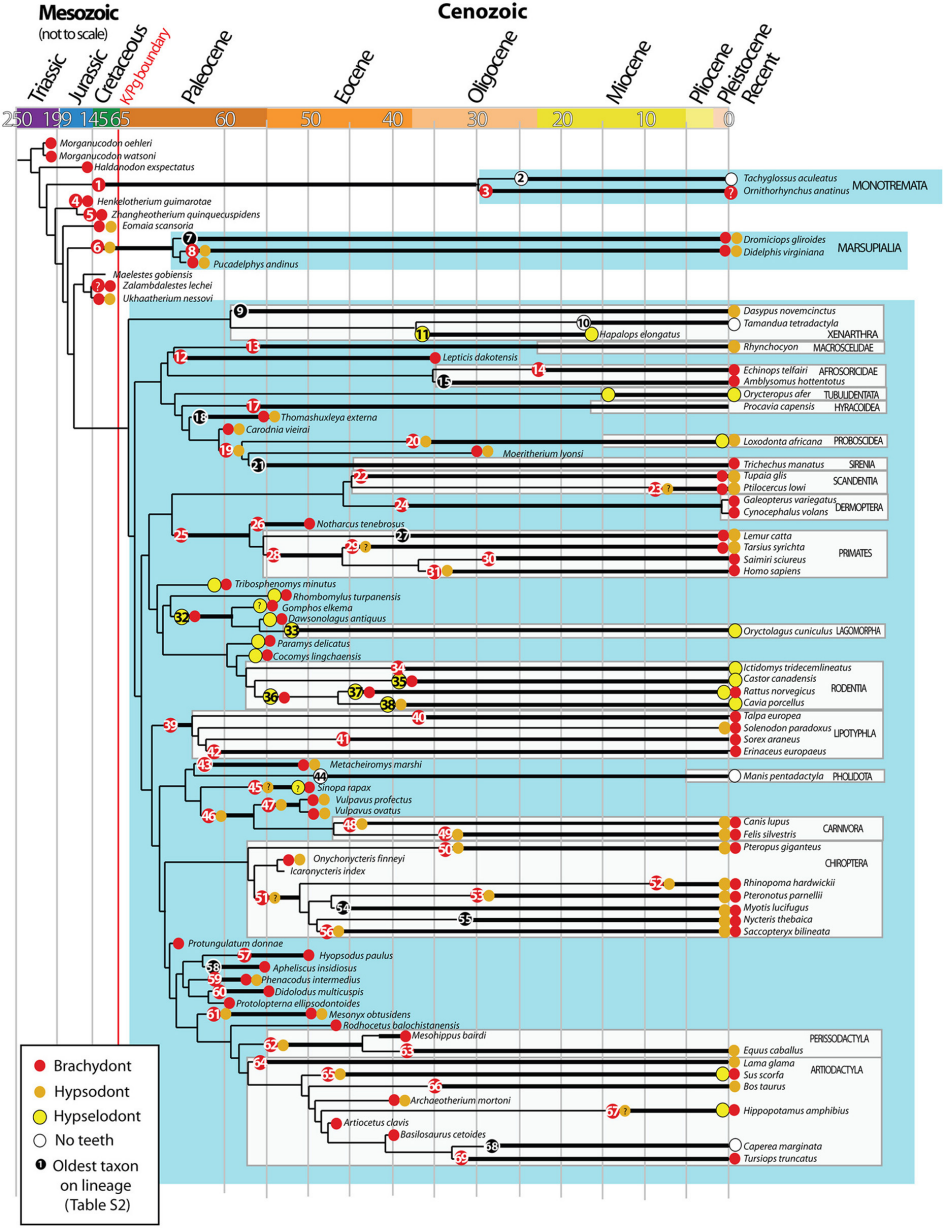
**Evolution of brachydonty, hypsodonty and hypselodonty in mammalian teeth through phylogeny**. Data from tooth types have been collected from the literature (see details in Table S2) and superimposed on the mammalian phylogentic tree performed by O'Leary et al. (2013). Node numbers refers to the oldest taxon on the lineage, reported in the Table S2. The colored circles indicate the tooth types associated to the species. Same color code as Figure 1. Black circles correspond to species without data on tooth types. ?: refers to unclear tooth type. Ages are in million years. Adapted from O'Leary et al. (2013).

In order to counteract the decrease in tooth number and the lack of tooth replacement capacity, some mammals developed hypsodont, i.e., high-crown, and hypselodont, i.e., ever-growing, teeth (Figures 1, 2; Tables S1, S2). Interestingly, the comparison of polyphyodont, diphyodont, and hypselodont dentitions can demonstrate the evolutionarily conserved potential of dental stem cells to form new dental tissue, from replacement teeth to the continuous growth of hypselodont teeth (Jernvall and Thesleff, 2012; Juuri et al., 2013). In this evolutionary scheme, whereas the number of teeth and their replacement tend to decrease in evolution leading to the reduced dental formula of rodents for instance, the tooth shape complexity increased and new strategies of tooth renewal appeared (Jernvall and Thesleff, 2012).

A tooth is considered as hypsodont if its crown height exceeds its length (or sometimes the width) (Van Valen, 1960; Janis and Fortelius, 1988). However, it is difficult to define precisely hypsodonty as mammalian teeth form a continuum between brachydont teeth (low crown and well-developed roots) to hypselodont teeth (ever-growing crown without roots). The intermediate stages consist in different degree of hypsodonty where the crown height might depend on a timing balance between crown production and root formation during the animal life span, i.e., heterochrony in ontogeny, as well as the wear rate for occluding teeth (Koenigswald, 2011). Hypsodonty can be defined also based on the nature of the biological tissues that compose the crown of the tooth and when these tissues develop during the ontogeny (Koenigswald, 2011). For example, crowns of hypselodont tooth are usually covered with enamel but some examples show only dentine-covered ever-growing teeth, such as elephants (e.g., *Loxodonta, Elephas*) incisors, the canines of the Indonesian babiroussa (*Babyrousa babyrussa*) and the walrus (*Odobenus*), and the molars of the sloth (e.g., *Bradypus, Choloepus*) (Table S2). The enamel present in juvenile teeth disappeared during the ontogeny of the animal. In addition, the asymmetric incisor of rodents, with enamel only on the labial side and while dentine is apparent on the lingual side, may correspond to a simultaneous and continuous development of the enamel and dentine tissue during ontogeny (Koenigswald, 2011).

Ever-growing teeth intrigued developmental biologists since the first description of continuously growing incisors of rodents by Waterhouse in 1848. However, over a century passed before the first experiments analyzing the phenomenon of tooth renewal were conducted (Hwang and Tonna, 1965; Smith and Warshawsky, 1975; Smith, 1980). These studies showed fast displacement of ameloblasts and odontoblasts in mice and rats, demonstrating rapid renewal of the incisors. The best example used to study ever-growing teeth is the mouse incisor. However, more studies nowadays are interested in ever-growing molars in other rodent models like the vole and the guinea pig (Tummers and Thesleff, 2003; Ohshima et al., 2005). In addition, hypsodont molars can be used to explore the mechanisms of the crown-root transition, meaning the level of hypsodonty during evolution.

This review aims to show how evolutionary biology gains from developmental biology to understand the evolution of this important phenotype in mammal evolution. At the same time, we demonstrate how developmental biology gains from evolutionary biology to observe the diversity and the conservation of the stem cell niche maintenance mechanism throughout geological time-scale.

We report for the first time, a review on the evolution of the crown height from all the tooth types in mammals from low-crown teeth, i.e., brachydont, to ever-growing teeth, i.e., hypselodont. We use fossil and recent data from the literature to determine a relative measure of the convergent appearances of hypsodonty and hypselodonty over geological times. We will see how the convergent evolution of high-crown teeth across placental mammals can be explained by developmental mechanisms that tinker with the main pathways of tooth development related with the maintenance of stem cell niches. We hypothesize whether the functionality of ever-growing teeth could have some consequences on the maintenance of the stem cell niche, or whether other developmental mechanisms could constrain the evolution of hypsodonty. The tooth renewal mechanism, which we will see, evolved several times in mammals, can be of great interest for tooth bioengineering in humans, as they both require the differentiation of stem cells during tooth development.

## Diversity and evolution of hypsodont and hypselodont teeth in mammals

Hypsodonty and hypselodonty are tooth types that evolved convergently through mammal evolution (Figures 1A, 2). This confirms the observations of convergent evolution of hypsodonty in mammalian orders solely with molars (Janis and Fortelius, 1988; Jardine et al., 2012). Even if most of the recent mammals retained the ancestral brachydonty (Figure 1A), both marsupial and placental mammals have developed hypsodont teeth, in all tooth types—incisors, canines, premolars, and molars (Figures 1A, 2). However, more placental orders have developed hypselodont teeth than marsupials (Figure 1A). Only one family in the marsupial order Diprotodontia, the Vombatidae, is known to show hypselodont teeth (Figure 1A; Table S1), while eight orders of placental have, at least, one tooth type which is hypselodont. In recent mammalian families, the incisor is more commonly hypselodont (24%) in comparison with the canines and the cheek teeth (premolars and molars). However, the incisor stays in majority brachydont (45%) (Figure 2B; Table S1). The canine is the tooth that shows the highest percentage of hypsodonty (24%), but half of the mammalian families have lost the canine during evolution (54%) (Figure 2B; Table S1). Cheek teeth are still in majority brachydont, but 18% of all the families show hypsodonty. Slightly more molars are hypselodont than premolars (Figure 2B; Table S1). While two orders of mammals have almost totally lost their teeth, the Monotremata (e.g., platyous) and the Pholidota (e.g., pangolin), the orders that show the highest diversity of tooth types are the Diprotodontia, the Artiodactyla, the Cetacea and the Rodentia (Figure 1). These last orders are highly diversified in terms of number of families as well (Table S1). However, that may not be the only explanation as the Chiroptera do not show a huge diversity of tooth types, only brachydonty and hypsodonty with no tooth loss, while this order is highly diversified. Only the order Cetacea has keratinous plates instead of teeth, e.g., the pigmy right whale (*Caperea marginata*) and in the order Sirenia, the Steller's see cow (*Hydrodamalis gigas*) have keratinous rostral pad in the front jaw (Figure 1; Table S1). One of the most intriguing specimens is the monotreme duck-billed platypus (*Ornithorhyncus anatinus*) which shows only molars made out of keratin while the juveniles have genuine brachydont molars covered by enamel (Ungar, 2010). Juveniles retained the ancestral state of enamel-covered brachydont molars (Musser and Archer, 1998; Grant, 2007) (Table S2).

To observe the evolution of hypsodonty in placental mammals, we assigned the different tooth types to all the extinct and recent mammals included in the most recent phylogenetic study from O'Leary et al. (2013). This study is invaluable as the phylogeny at the lineage level, from fossil to recent species, allows us to follow the evolution of hypsodonty precisely. Even if the phylogeny of O'Leary et al. (2013) does not cover all the diversity of mammals, especially the marsupial mammals, we managed to confirm that hypsodonty and hypselodonty are convergent phenotypes that appeared at different time periods during evolution (Figure 2; Table S3; Figure S1).

During evolution, all the tooth categories have the tendency to become less and less brachydont (Figure S1), but it is still the majority in all teeth of placental mammals (Figure 1B). The incisors started to become either hypsodont, either hypselodont during the mammalian radiation at the beginning of the Cretaceous (Figure 2; Table S2). The increase of hypselodonty in incisors at the beginning of the Paleocene (c.a. 64 Ma) and during the Eocene correspond mainly to the Rodent radiation, while the increase of hypsodont incisor during the Eocene–Oligocene may be more related with elephant fossil relatives (*Moeritherium lyonsi*, Delmer et al., 2006 and *Barytherium grave*, Delmer, 2009; Table S2). The sample size of the Oligocene and the Miocene species are unfortunately too low in the dataset from O'Leary et al. (2013) to draw evolutionary interpretations (Table S3; asterisks in Figure S1). However, we can clearly observe that canines became drastically less brachydont during the Cretaceous, the Paleocene and the Eocene and have been replaced by hypsodont canines (Figure 1B; Table S3; Figure S1). Most of the hypsodont canines are present in the orders Carnivora and Chiroptera and their ancestors, but some Artiodactyla have hypsodont canines as well (Tables S1, S2). What characterizes the evolution of canines is the increasing number of mammal species that lost their canine during evolution (Figures 1A,B; Figure S1). These species are mostly plant eaters like the Anthracotheriidae, the Bovidae, the Equidae, the Rodentia and Lagomorpha, including their ancestors (Figure 2; Table S2). It does not mean that carnivores didn't lose any teeth during evolution, and actually they did, especially first premolars and distal molars, but we cannot see it from this dataset because we are separating tooth categories, and the canine is the only tooth in its category. So, when the canine disappears, it becomes very drastic in this dataset. Hypselodont canines appeared more recently during mammalian evolution; a few modern mammals display hypselodont canines, such as the wild boar (*Sus scrofa*) and the hippopotamus (*Hippopotamus amphibius*) (Figure 1B; Table S2). Premolars and molars evolved hypsodonty and hypseldoonty almost at the same time during the Eocene, but most of the teeth stayed brachydont (Figure S1). Nevertheless, hypsodont and hypselodont premolars and molars appeared much later in evolution in comparison with incisors and canines (Figure S1).

According to the dataset of O'Leary et al. (2013), there are no reversals observed (i.e., (Dollo, 1893): “*An organism cannot return, even partially, to a previous state already realized in its ancestral series*”). It means that when the hypselodonty is established in one lineage, it might never return back to a hypsodont tooth. The same seems to be true from hypsodonty to brachydonty. However, this dataset is not complete enough to represent all the mammals tooth evolution, and it may be possible that other lineages showed reversion of tooth type, even if this evolutionary mechanism is rare.

## Epithelial stem cells in hypselodont teeth

### Dental stem cell location

While hypselodonty appeared several times during mammalian evolution, continuously renewing teeth are mainly studied in rodents, and more precisely in mouse and rat, due to their prevalence in laboratories as animal models. What we will describe here resulted from studies on the incisor of murines.

Similar to other renewing organs, such as intestine (Snippert et al., 2010), the homeostasis of the murine incisor is maintained by stem cells localized at the base of the tooth in a structure called cervical loop (CL) (Harada et al., 1999). Their role is to renew the tip of the tooth that is lost by attrition. Although the study of incisor stem cells is a relatively new field, significant advances have been made in the identification of these cells, the understanding of their functions, and the characterization of molecular mechanisms regulating their dynamics.

Tooth renewal capacity relies on epithelial and mesenchymal stem cells that give rise to the different cell lineages of the tooth. While dental mesenchymal stem cells differentiate into dental pulp cells and odontoblasts, secreting dentine, dental epithelial stem cell differentiation gives rise to several cell type, among them are the ameloblasts, producing enamel. The murine incisor displays on its labial side a layer of enamel. Therefore, this side is called crown-analog, as opposed to the lingual side, enamel-free and covered with dentine, hence called root-analog.

**Figure 3:**
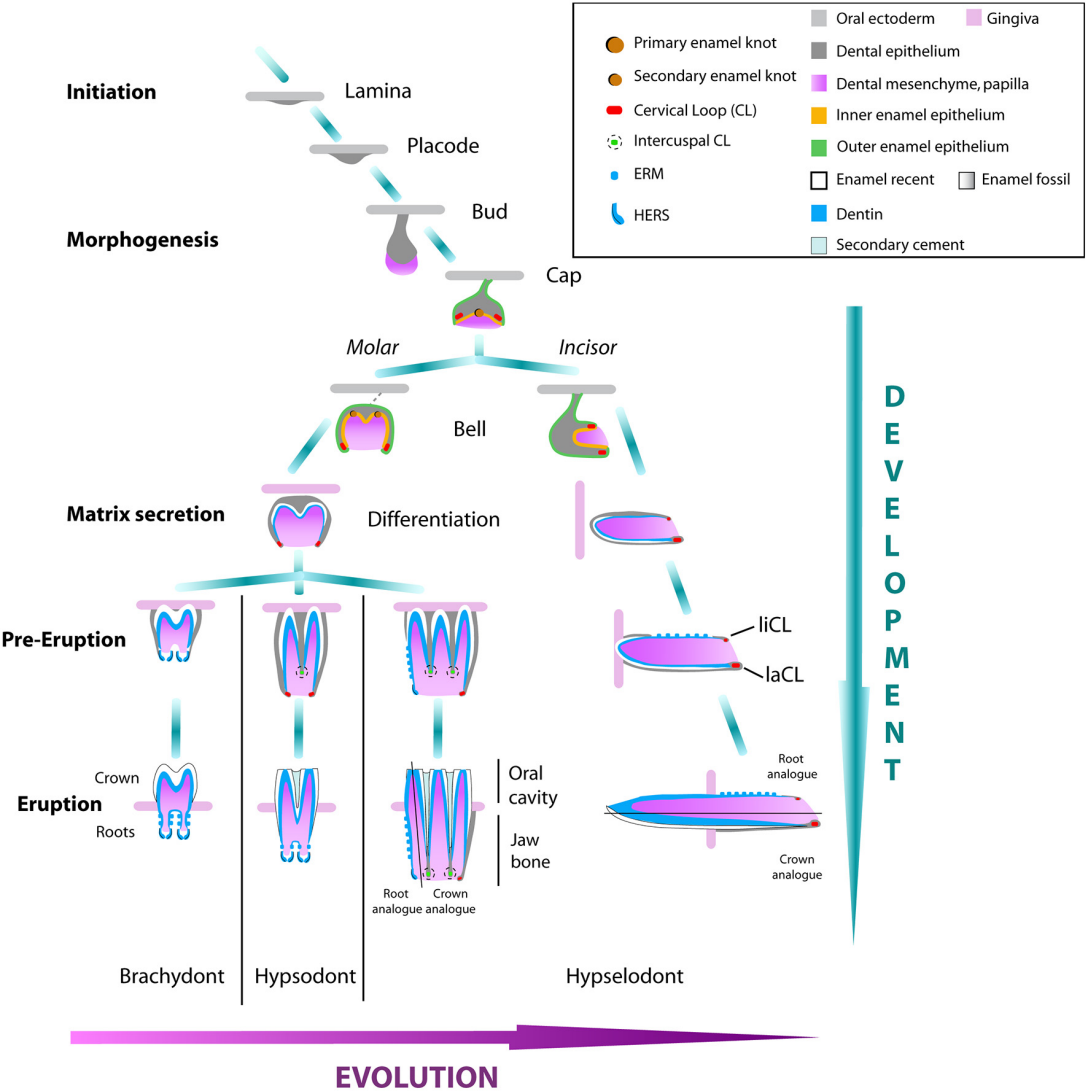
**Tooth development of molars and incisors in rodents from brachydont to hypselodont teeth**. The initation stage of tooth development starts with the formation of the dental lamina. The epithelium signal attracts the underlying mesenchymal cells that condense into a placode. The responsive epithelial cells start to introvert into the mesenchyme forming the bud stage. The formation of the first enamel knot starts the cap stage. Multicuspid molars, or premolars, will differentiate into the secondary enamel knot from the first enamel knot, while the unicuspid incisor grows antero-posteriorly from the labial and lingual CLs (liCL and laCL) at the bell stage. During the differentiation stage, epithelium-derived ameloblasts and mesenchymal-derived odontoblasts will secrete a cell matrix that will form the enamel and the dentin tissues respectively of the tooth crown. At early eruption, in the brachydont molars, the CLs close and differentiate in the HERS cells that start to form the roots and will continue to grow during the life span of the animal. The activity of the CLs forming the crown of hypsodont molars, will stop much later in development and this timing is different depending on the mammal species. Hypselodont molars and incisors will never form proper roots, but will form an asymmetry between a root analog and a crown analog. Modified from Tummers and Thesleff (2003); Jernvall and Thesleff (2012).

**Figure 4:**
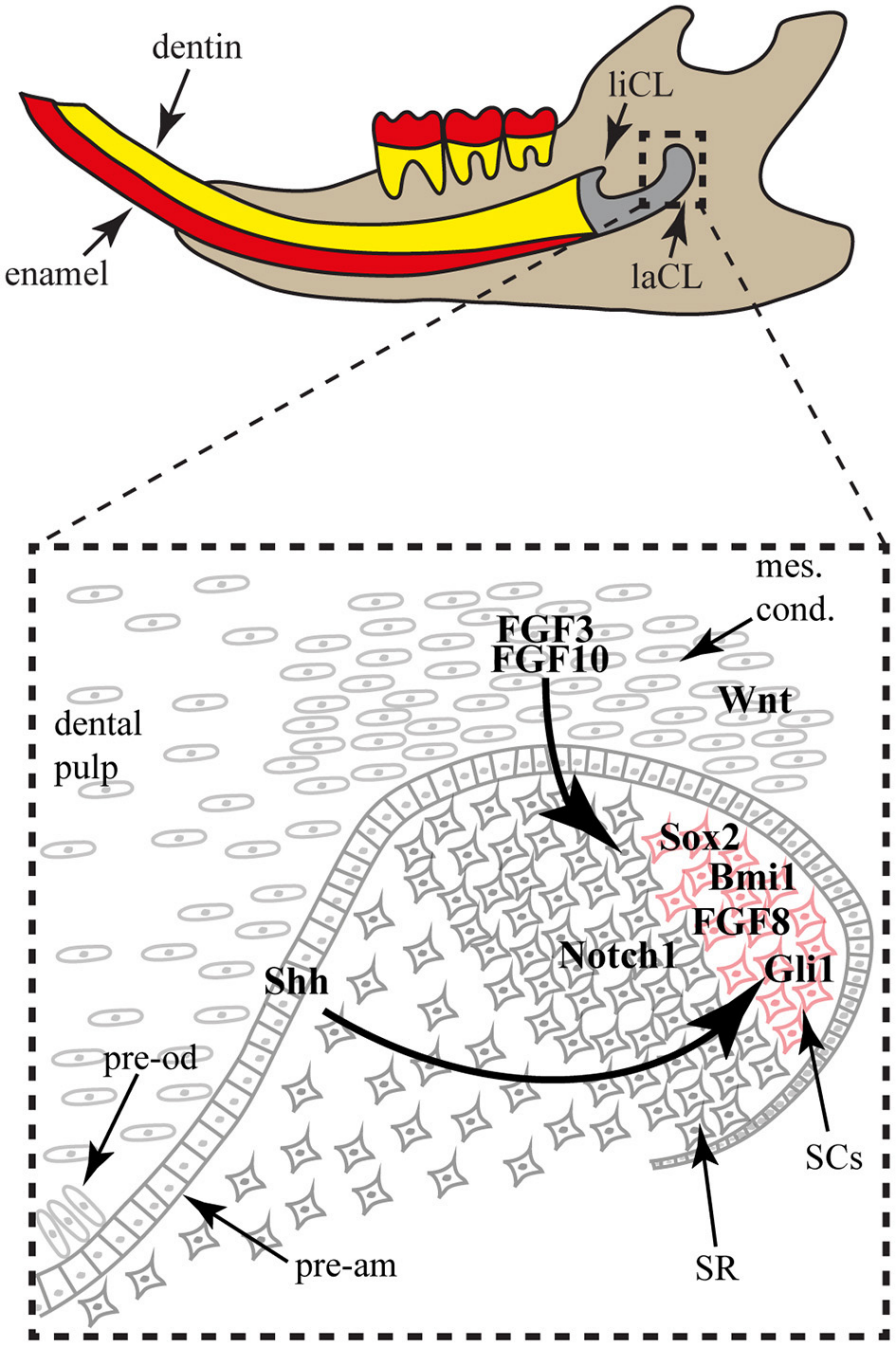
**The mouse incisor stem cell niche**. The lower incisor is shown in sagittal view in the jaw. The proximal structure is composed of the lingual (liCL) and labial (laCL) cervical loops, housing the stem cell populations. In the laCL (close-up), the stem cells (SCs) are next to the stellate reticulum (SR) cells. When the SCs divide and differentiate, they exit the niche to be integrated in the enamel epithelium and form pre-ameloblasts (pre-am). Around the epithelial niche a mesenchymal condensate (mes. cond.) contains mesenchymal stem cells that will give rise to the dental pulp and the pre-odontoblasts (pre-od). The mesenchymal expression of FGF3 and FGF10 is important for the SC maintenance. While the SR cells express Notch1, the SCs express FGF8, Bmi1, Gli1 and Sox2. Gli1 expression is part of a feedback loop induced by the Shh expression by the SC early progeny.

*In vitro* culture experiments demonstrated that the cells fueling the continuous growth of the rodent incisor were housed in the two cervical loops (CLs) located at the base of the tooth (Harada et al., 1999). Therefore, while the lingual CL renews the root analog of the incisor, the labial CL renews the crown analog (Figures 3, 4). The stem cells in the labial CL give rise to progenitor cells that undergo several rounds of cell division before differentiating into ameloblasts (Figure 4; Thesleff and Tummers, 2008). After reaching the enamel epithelium, the epithelial cells are no longer actively migrating, but are pushed, or displaced, toward more differentiated areas in a mechanism similar to a conveyor belt. The lingual CL is logically thought to house the stem cells that are required for renewal of the root analog surface of the incisor. However, this structure has been less studied than the labial CL, and thus requires further examination to determine its function.

While label-retaining approaches allowed the identification of slow dividing cells in the adult mouse labial CL (Harada et al., 1999; Seidel et al., 2010), only lineage-tracing experiments brought knowledge on specific SC population dynamics and organ renewal. For instance, the lineage tracing of the Gli1+ cell population demonstrated the involvement of these cells in the long-term renewal of the incisor epithelial compartments (Seidel et al., 2010). More recently, two other cell populations localized in the labial CL were shown to participate in incisor renewal via genetic fate mapping, namely Sox2+ and Bmi1+ cells (Juuri et al., 2012; Biehs et al., 2013). Interestingly, the Sox2+ cells, Bmi1+ cells and Gli1+ cell populations (Figure 4) might not completely overlap and seem to have different dynamics during tooth homeostasis, reflecting a possible hierarchy of stem cells in the hypselodont tooth renewing.

Besides the lineage tracing experiments, some factors are known to be expressed by cells located in the Sox2+ and/or Bmi1+ area (Figure 4), such as Lgr5 (Suomalainen and Thesleff, 2010), Yap, ABCG2, and Oct-3/4 (Li et al., 2011).

While the recent genetic fate mapping experiments are giving definite answers regarding the identity of the incisor SCs, the molecular cues regulating SC maintenance and tooth homeostasis was under close scrutiny for several decades already.

### Molecular regulation of stem cell maintenance

Over the past 35 years, the roles of the principal morphogenetic signaling pathways in tooth development have been studied, leading to a deep understanding of the functions of these pathways (Thesleff et al., 1979). Interestingly, the pathways involved in ectodermal organ morphogenesis are also involved in stem cell regulation for organ renewal. Discoveries of numerous actors of conserved signaling pathways have strengthened our understanding of the genetic networks regulating tooth formation and renewal (Tummers and Thesleff, 2009).

While most of the conserved morphogenetic pathways, such as Notch, Bmp, Wnt and Shh, were shown to play a role in incisor morphogenesis and homeostasis, the conserved and well-known Fgf pathway became particularly interesting in regards to stem cell maintenance and differentiation (Harada et al., 1999, 2002; Parsa et al., 2010). More specifically, *Fgf3* and *Fgf10* are expressed in the mesenchymal cells surrounding the labial CL (Figure 4), whereas *Fgfr1b* and *Fgfr2b* are enriched in the stem cell niche. The use of genetically modified animals helped to elucidate the mechanism of Fgf pathway involvement in the stem cell niche. While the incisors of *Fgf3* null newborn mice are normal in comparison with wild-type mice, double mutation of Fgfs (*Fgf3^−/−^; Fgf10^+/−^*) display incisors with drastic hypoplasy in the labial CL, reflecting the importance of Fgf signaling in the maintenance of the incisor stem cells (Wang et al., 2007). A similar phenotype was obtained upon reducing Fgf receptor function, using a tetracycline-inducible FGFR2b dominant negative system (Parsa et al., 2010).

Moreover, modifying factors interacting directly with the Fgf pathway have shown drastic phenotypic modifications of the incisor CLs size and stem cell maintenance. The sprouty genes, targets and negative regulators of Fgf signaling, are expressed in and around both labial and lingual CLs. Due to the loss of sprouty function, the lingual CL was enlarged and morphologically similar to the labial CL. Even ameloblasts differentiated on the root-analog side of the incisor (Klein et al., 2008). While ectopic expression of *Follistatin* led to reduced mesenchymal *Fgf3* expression and a severely hypoplastic labial CL. Conversely, the epithelial loss of *Follistatin* expression induced up-regulation of *Fgf3* expression in the mesenchyme adjacent to the lingual CL, causing, similarly to sprouty inhibition, an expansion of the lingual CL and a differentiation of ameloblasts on the lingual side (Wang et al., 2007). Inactivation of the *Alk5*/*Tgfbr1* receptor in the mesenchyme led to down-regulation of *Fgf3*, *Fgf9*, *Fgf10* and reduced the size of the labial CL, likely due to cell proliferation defects (Zhao et al., 2011). Interestingly, Fgf8 activity was shown to induce *Sox2* expression and therefore playing a direct role on stem cells (Figure 4; Juuri et al., 2012). Several lines of evidence indicate that Fgf signaling is involved in the maintenance of the incisor stem cell number and therefore, to maintain the asymmetric production of ameloblasts on the labial side through a smaller subset of cells within the labial CL (Figure 4).

While Fgf signaling is undoubtedly important for incisor stem cell maintenance, Wnt signaling seems to require inhibition rather than activation during incisor renewal. While this pathway is active during tooth morphogenesis (Suomalainen and Thesleff, 2010), several Wnt inhibitors are expressed in the labial CL and no Wnt activity was detected in the stem cell niche (Figure 4; Juuri et al., 2012). Moreover, the over expression of *Wnt3* in the dental epithelium led to a progressive loss of ameloblast differentiation during incisor renewal (Millar et al., 2003).

Like other renewing organs, stem cell differentiation in the hypselodont tooth is controlled by factors expressed within the stem cell niche and by exogenous factors. In order to maintain tissue homeostasis, the stem cell progeny should replace the cells lost to wear and tear. The Hedgehog signaling pathway was recently shown to be involved in the feedback signals from the differentiated cells to the stem cells. *Shh*, expressed by the progeny of differentiating cells, signals back to the progenitor stem cells through a positive feedback loop in order to produce more progeny (Figure 4; Seidel et al., 2010). Interestingly, another Hh-responsive cell population is located in the mesenchyme, and is constantly producing new odontoblasts producing dentine.

### Crown-to-root transition: loss of stem cell maintenance

To build and maintain a tooth, a balance must be struck between supplying cells to the crown which functions in eating, or to the root that is critical to holding the tooth in place. This balance is known as the crown-to-root transition and the variation that exists is exhibited by the level of hypsodonty from brachydont teeth to hypselodont teeth. While, from the evolutionary perspective, hypsodonty and hypselodonty results from a retardation, or absence, of root formation during ontogeny, from the developmental perspective, hypsodonty resembles an exhaustion of stem cells leading to a late crown-to-root transition in rooted teeth. This transition is characterized by the loss of stellate reticulum cells in the CLs, marking the end of the crown morphogenesis that leads to the formation of the roots (Tummers and Thesleff, 2008).

At the time of the crown-to-root transition in the murine molar, the loss of epithelial Notch and mesenchymal Fgf signals were reported, whereas vole molars continue to express these key signaling components and bypass root fate (Tummers and Thesleff, 2003). This observation was confirmed when the *in vitro* modulation of Fgf signaling was achieved via up-/down-regulation of FGF10, leading to the continuous growth of the murine molar crown (Harada et al., 2002; Yokohama-Tamaki et al., 2008). Regulation of the crown-to-root transition is also achieved during murine incisor morphogenesis. For instance, the progressive loss of the stellate reticulum cells in the lingual CL is necessary for the formation of the root analog. The analysis of murine mutants, exhibiting either 2-sided crown analogs or 2-sided root analogs, provides clues regarding the mechanisms of crown-to-root transition. Several murine mutants are known to develop such defects in incisor asymmetry, including *Fst* and *Sprouty* nulls (Wang et al., 2004; Klein et al., 2008), as well as *Krt14*-driven overexpression of *Fst* and *Eda* (Mustonen et al., 2003). Although correlative, these studies suggest that molecular cues govern stem cell maintenance, which differentiate brachydont, hypsodont and hypselodont teeth.

## Discussion

### Tinkering with the stem cell niche and diversity of hypselodont teeth

The evolution of tooth hypsodonty and hypselodonty, do not necessarily require the creation of new genes or new genetic pathways (Keränen et al., 1998; Tummers and Thesleff, 2009). Pathways in tooth development are generally evolutionary well conserved across vertebrates (Jernvall and Thesleff, 2012). For instance, the same signaling pathways are required in brachydont, hypsodont or hypselodont tooth development (Tummers and Thesleff, 2003). The biologist Jacob (1977) first exposed the concept of “*tinkering*” with development, saying that development takes what already exists to generate different forms by re-organizing the network connections (Jacob, 1977 in Laubichler and Maienschein, 2007). Experimentally, tinkering with the regulation of genes involved in murine incisor stem cell regulation can transform the ever-growing incisor, displaying an enamel-dentin asymmetry, into a double sided-enamel or enamel-free tooth (see the Section Molecular Regulation of Stem Cell Maintenance). These various phenotypes mimic hypselodont teeth that appeared during evolution, such as the thick single-sided enamel incisor of the aye-aye (*Daubentonia madagascariensis*), the double-sided enamel canines of the Korean musk deer (*Moschus moschiferus*) or the wild boar (*Sus scrofa*), the enamel-dentine asymmetry of all the Glires incisors and the enamel-free incisor of the Elephantidae. Basically tinkering with stem cell regulation would generate all the uniscupid hypsodont phenotypes described by Koenigswald (2011): (1) the “enamel-band hypsodonty” where the enamel is mostly, if not totally, covering the tooth, (2) the “unicuspid hypsodonty” where the teeth are growing “freely” without wearing by occlusion and (3) the “dentine hypsodonty” where the dentine is mostly, if not totally, covering the ever-growing tooth. For Koenigswald, these categories of hypsodont teeth are dependent on the expansion of one tissue growth over another tissue during ontogenetic phases (see Introduction). However, we know from the developmental studies (see Section Epithelial Stem Cells in Hypselodont Teeth) that the formation of enamel and dentine are always synchronous during development. It is more the size of the CLs, the nature, the number, the maintenance and the spatial regulation of the stem cells within the CLs that will determine the nature of the tissues that will be secreted (odontoblasts and/or ameloblasts) during ontogenetic stages. The heterochrony of the ontogenetic stages defined by Koenigswald, might have a great importance in defining the crown-to-root transition during tooth development, but it seems not to be the main mechanism defining the nature of the tissues that will characterize the diversity of hypsodont and hypselodont teeth.

We are lacking experiments on embryonic premolars and molars to define if tinkering with stem cell regulation in CL would generate the “multicuspid hypsodonty” or the “sidewall hypsodonty” (i.e., multicuspid hypsodont teeth, enamel-covered with or without dental tracts) defined by Koenigswald (2011). However, it is doubtful that solely the wearing of the teeth, and/or the time changing of the tissues growth during ontogeny would be responsible for the spatial distribution of enamel and dentine along hypsodont and hypselodont teeth.

Hypselodont teeth evolved at different time during mammalian evolution and in different species. This convergent evolution of the hypselodont teeth cannot explain whether the size of the stem cell niche evolved toward a reduction, and then an enamel-free tooth or from an enamel-free tooth to a double-sided enamel tooth, through an intermediate asymmetric enamel-dentine tooth. No real evolutionary tendencies can be found here, but rather an exploration of every possible kind of tooth, tinkering with the stem cell niche during ontogeny and evolution.

### Is the evolution of hypselodonty only dependent on the capacity to maintain the stem cell niche during ontogeny?

During tooth evolution, the transition from a brachydont to a hypsodont tooth is thought to have led to the formation of the hypselodont tooth. Hypsodonty results from an extended crown growth period fueled by a transitory maintenance of stem cells located at the base of the crown. This results in a higher crown compared to the brachydont teeth. A life-long persistence of the stem cells was suggested as the cause for the appearance of hypselodont teeth (Tummers and Thesleff, 2003).

Hypsodonty is therefore the best example of the essential role of heterochrony in ontogeny. The timing of crown-to-root transition during development, which is associated with the depletion of the stem cells in the CL, varies greatly between species and determines the degree, or level, of hypsodonty, during evolution (Fortelius and Solounias, 2000). The paleontologist Stephen J. Gould has first defined the concept of “heterochrony in ontogeny” when he said, “*If development stages are retarded or accelerated while size and shape remain in their ancestral relationship, we observe heterochrony in evolution*” (Gould, 1977). He used this concept to define the mechanisms that lead to evolutionary trajectories in animal morphologies. We have seen with the hypsodonty example, that the heterochrony in ontogeny, is more related with the capacity of the dental epithelium to differentiate into enamel-producing ameloblasts during crown development. Therefore, the heterochrony is a good mechanism to differentiate tooth types between brachydont, hypsodont and hypselodont but not to categorize teeth based on their tissue types (enamel, dentine, cement) as suggested by Koenigswald (2011). The key mechanism is then to understand what regulates the timing of the crown-to-root transition. The developmental mechanism maintaining hypselodonty is another interesting question, i.e., what happens when a hypsodont tooth acquires the capacity to grow continuously. Thus, the developmental timing of the crown-root transition represents a key feature in the understanding of the heterochronic evolution of hypsodonty and hypselodonty in mammals.

According to Janis and Fortelius (1988), “*it is evident that in a […] tooth the transmission of occlusal stress from tooth to bone will pass through the base of the root or, in the case of continuously growing teeth, through the area of secretion.*” The area of secretion would correspond here to what we know now as the stem cell niche. We hypothesize that the physical forces of occlusion, dependent on the animal diet, its volume, soil grit and/or tooth attrition, might have a mechanical effect on the tooth, and in turn, affects the stem cell niche, through a feedback loop pathway like Shh, that may delay the crown-to-root transition during ontogeny. This hypothesis assumes that there are crown height variations within and between individuals and possible reversions during evolution, and it assumes as well that an occlusion between teeth is necessary to maintain the crown growth in hypsodont and hypselodont teeth. However, we didn't observe any reversions in our dataset, the crown height variations within individuals are hard to observe, in large mammals for instance, and ever-growing teeth can be maintained without any occlusion (i.e., Elephant tusks). In addition, if we consider the strong heritability of the degree of hypsodonty in large mammals, shown by Raia et al. (2011), then this assumption is false. Nevertheless, the observation of a high variation in the crown height within the large and small mammals populations at the beginning of the adaptive radiation, then a decrease of this variation during evolution, might favor this hypothesis. In fact, an external factor that would impact on the delay of maintenance of the stem cell niche can be a variable trait at the beginning of the mammalian radiation, which we observed from the Figure 2, and then it becomes more stable within lineages and becomes a heritable trait independently in different orders (Figure 1), without reversions. The developmental plasticity of the crown height at the population level might have favored the evolution of hypsodonty, and then hypselodonty within mammalian lineages (Moczeck et al., 2011). That could explain why hypsodonty and hypselodonty are phenotypes that can appear in different mammalian clades, at different time of the mammalian radiation.

So far, none of these difference in hypsodont variability have been observed at the level of mammalian populations, but the potential relationship between the stemness capacity, the level of hypsodonty and the environmental cues that stimulate cell division and differentiation might resolve the mechanisms that correlate the convergence of hypsodonty in teeth in the fossil records.

Hypselodonty in molars is quite rare in large mammals. For instance, the rhinocerotidae *Elasmotherium*, from the Miocene (Antoine, 2003), and some species of Xenarthra (Jardine et al., 2012) shows ever-growing molars and/or premolars. Most of the herbivorous mammals have hypsodont premolars and molars at different degree of crown height, but mainly remaining a root formation even at late stages of the animal ontogeny. Janis and Fortelius (1988) explained the paucity of hypselodont large mammals in comparison with hypselodont small mammals, by a developmental constraint in the enamel formation. For these authors, the ungulates molars present enamel pits within the crown (isolated fossae) that may be present before the tooth eruption, which would prevent the tooth to become hypselodont without a substantial reorganization of the structure of ameloblasts-secreting epithelial cells. In small mammals, an equivalent structure could be the “enamel islet,” which is found in the fossil *Mimomys* lineage for instance, in the recent cricetids *Neotomodon* and *Neotoma*, which has hypsodont teeth (Hillson, 2005). This enamel islet has been lost in the evolution for most of the Arvicolines (Chaline et al., 1999), and most of them developed hypselodont molars. The genus *Myodes*, however, still have hypsodont molars, but without enamel islet. Therefore, the crown-to-root transition in small and large mammals could be due to physical properties of the secreted tissues and a spatial reorganization of the stem cells secreting ameloblasts in the CLs during tooth ontogeny, and not only a time-changing of the stem cell maintenance.

### Hypsodonty and environment: lamarckism, darwinism, both?

“[…], *the teeth of some of the animals have one function only, to break up the food. Of those animals whose teeth serve also as a defense and as weapons, some (like Swine) have tusks, some have sharp interlocking teeth, and are called “saw-toothed” as a result. […]. These teeth are used in self-defense by biting; tusks by stricking. This explains why sows bite: they have no teeth*.” Aristotle (-384, -322) (Translated by Peck and Foster, 1983; P. 211).

Hypsodont canines evolved mostly into weapon function to grab preys in carnivore predators (Van Valkenburgh, 2007), but as well in sexual dimorphism by the sexual selection of canines in primates for instance (Plavcan, 2012). Hypselodont canines, and/or incisors without occlusion, i.e., “free growth” according to Koenigswald (2011), would more correspond to defense function, like the ever-growing canines of the walrus (*Odobenus rosmarus*), or the babiroussa (*Babyrousa babyrussa*) and the elephant (*Loxodonta* and *Elephas*) incisors. However, it seems that the evolution of these phenotypes would be more related with the available preys and the behavior than direct environmental cues.

Scrutinizing the diet evolution of hypsodont molars in herbivorous large mammals during climate change has shown that hypsodonty might allow an enlargement of the dietary range within herbivores, than a restriction to abrasive grasses (Feranec, 2003; Kaiser, 2003; Mihlbachler et al., 2011). Therefore, the development of hypsodonty in large mammals, and especially in ungulates, might have favored the populations to spread out in open landscape while the Miocene climate changed toward drier conditions, than local adaptation to changing vegetation (Fortelius et al., 2014). It seems likely that high-crown teeth would be adapted for abrasive diet, but it rather allow the animals to get a broader range of food that might have enabled them to colonize more niches during the Neogene (Fortelius et al., 2014).

In the same way as large mammals, hypsodonty and hypselodonty has often been described in herbivorous small mammal as an innovation in the context of important environmental changes (Chaline et al., 1999; Vianey-Liaud and Michaux, 2003; Perez and Vucetich, 2011). However, the mechanisms that favored the evolution of incisors first and then molars toward hypselodonty in rodent lineages are not known. Ever-growing incisors are known for gnawing mostly, but can serve as defense weapons, as observed by Aristotle already. For Arvicolines, the dispersion dynamic was dependent on major climatic events (Tougard et al., 2008; Renvoisé et al., 2012) even if we can hypothesize that small mammal population spread out less than large mammals, and then they needed to adapt more to local changing environment than large mammals. Hypsodont and hypselodont molars are frequent in fossorial rodents as described in the evolution of the rodent lineages of American Great Plains at the Oligocene-Miocene transition (Jardine et al., 2012). Becoming semi-fossorial, fossorial, or even subterranean, could be one strategy for some small mammals to protect from predators in open environments (Ebensperger and Blumstein, 2006). These small mammals usually show chisel hypselodont incisors specific for digging in different soil textures (Becerra et al., 2014). Therefore, dust and grit ingestion for fossorial mammals could have an effect on the appearance, on the selection, and/or on the development of hypsodonty in small mammal lineages without strong relationship with the animal diet, but rather with the long-lasting properties of the tooth to compensate from wearing. Additionally, hypsodont molars in herbivorous small mammals could favor, as in large mammals, a broaden diet range. In small mammals, like the herbivorous voles with hypselodont molar, the *Microtus* genus, have a larger food range and are more opportunistic for their diet than *Myodes* or *Myopus* genera (Rinke, 1991).

This counterintuitive observation in large and small mammals is explained by the theoretical scenario known as the Liem's paradox (Fortelius et al., 2014). As hypothesized by Karel Liem (1980) in Cichlid fishes, “*In functional and constructional morphology, adaptation should be considered not as the response of a trait to external conditions but as its response to the total ensemble of the external environment.*” In other words, it would mean that a specific morphological trait, within an animal population, may not be “adapted” to specific environments, but is rather useful in a broader range of environments. Animals often avoid the foods to which they are morpho-functionally adapted (Robinson and Wilson, 1998).

As we discussed earlier, it seems that during tooth evolution brachydonty, hypsodonty, and hypselodonty were tried for all tooth types. However, it is difficult to know if the modulations of the duration of stem cell maintenance have been positively selected because of a constrain change from animal environment, or if the change in the animal environment led to a modification of the stem cell maintenance. In the first case a Darwinian inheritance would favor the most fitted, while in the second case we would assume that a Lamarckian inheritance would lead to the appearance of new features.

Evolutionary biologists have the tendency to favor Darwinian inheritance, however more and more data are suggesting that a Lamarckian inheritance occurs in several contexts. In their recent work, Pisco et al. (2013) demonstrated that on the cellular level, a leukemic cell can acquire a drug resistance by Lamarckian induction. In another example, Dias and Ressler (2014) gave evidence of a parental olfactory experience that can influence the subsequent generations. In this case, the modifications of the olfactory receptors are already observed in the first generation (F1). These results are of interest for tooth evolution. While environment has been seen as a tool shaping tooth evolution by the Darwinian selection of individuals able to subsist on a broader diet, maybe the climate change led to a rapid modification of tooth phenotype because of parental experiences.

According to Harada et al. (1999), stem cells respond to environmental cues that stimulate cell division and their cell progeny. Likewise, Tummers and Thesleff (2009) concluded that “*the stemness of epithelial cells does not seem to be determined intrinsically, but is regulated by the environment as is also shown by the tinkering of the stem cell niche properties in the incisor*.” These authors talked about local environment during development, such as the surrounding mesenchymal cells and signals. However, could epigenetic factors, environmental factors, or other external factors play a role in changing the fate of the stem cells? This key evolutionary and developmental question is yet to be understood.

## Conclusion

Understanding the switch from brachydont to ever-growing tooth might remain challenging as the steps and molecular modifications leading to the selection of specific features can only be hypothesized. However, the appearance and maintenance of dental stem cells offers an interesting model to study how dental cells can differentiate. This is the first step to achieve the formation of a tooth *in vitro*. However, much work remains to be done to achieve potential applications in regenerative medicine.

Before developing such protocols, some more basic questions should be answered first. For instance, the homing and segregation of the dental stem cells remains so far unknown. This process surely involves a complex molecular network missing in brachydont teeth. The genomic evolution of the factors involved in the dental stem cell homing might give new hints on the evolutionary process that has lead to the hypselodont tooth appearance. Interestingly, the true nature of the dental epithelial stem cells is still not known. Moreover, several stem cell populations co-exist in the CL. Therefore, more studies should focus on the dental stem cell dynamics before using them *in vitro*.

When these remaining questions will be answered, the development of bioengineered teeth will get easier. Two approaches are favored regarding the development of a biological tooth implant applicable in regenerative medicine: (1) growing a complete new tooth inside the jaw or (2) culturing the proper cell populations on a scaffold. While both approaches seem to be perfectly valid and already tested in animals (for review see Otsu et al., 2014), the main issue remains the source of cells to be used.

The first approach requires epithelial and mesenchymal cell populations with dental identity that are able to interact with each other in order to recapitulate the steps of tooth formation. The second approach requires progenitor cells able to colonize the scaffold and give rise to the various cell compartments of the tooth. Therefore, finding the role of each naïve cell population involved in odontogenesis and the manner in which cells differentiate is crucial to building a biological tooth implant.

The remaining challenges, together with the increasing number of tools available, make this an interesting time to study the Evo-Devo of tooth types and dental stem cells.

### Conflict of interest statement

The authors declare that the research was conducted in the absence of any commercial or financial relationships that could be construed as a potential conflict of interest.
