# Supplementary material for: An Evo-Devo perspective on ever-growing teeth in mammals and dental stem cell maintenance
Source: Front Physiol. 2014 Aug 28;5:324. doi: 10.3389/fphys.2014.00324 (PMC4147235; doi:10.3389/fphys.2014.00324)
Supplement: Supplementary file 1 [file DataSheet1.DOCX]

**Table S1: Diversity of Recent mammals for the different tooth categories – brachydont, hypsodont, hypselodont and no teeth – at the Order and Family level**. The Orders and Families are defined from Musser and Carleton (2005). The names are sorted based on the Figure 1. b = bunodont; h = hypsodont; hl = hypselodont; l = lower jaw; nt = no tooth; p = plates.

| **Order** | **Family** | **Incisor** | **Canine** | **Premolars** | **Molars** | **Sources** |
| --- | --- | --- | --- | --- | --- | --- |
| Monotremata | Tachyglossidae | nt | nt | nt | nt | 38 |
|  | Ornithorhynchidae | nt | nt | nt | b? | 38 |
| Paucituberculata | Caenolestidae | h (l), b (u) | b | b | b | 38 |
| Didelphimorphia | Didelphidae | b | h | b | b | 38 |
| Microbiotheria | Microbiotheriidae | b | h | b | b | 38 |
| Notoryctemorphia | Notoryctidea | b | b | b | b | 38 |
| Dasyuromorphia | Thylacinidae | b | h | b | b | 38 |
|  | Myrmecobiidae | h? | h | b | b | 38 |
|  | Dasyuridae | b | h | b | b | 38 |
| Peramelemorphia | Thylacomyidae | b | h | b | b | 38 |
|  | Chaeropodidae | b | b | b | h (l), b (u) | 38 |
|  | Permelidae | b | b | b | b | 38 |
| Diprotodontia | Phascolarctidae | h | b (u), nt (l) | b | b | 38; 37 |
|  | Vombatidae | hl | hl | hl | hl | 38; 37 |
|  | Phalangeridea | h (l), b (u) | b | b | b | 38; 37 |
|  | Petauridea | h (l), b (u) | b | b | b | 37 |
|  | Hypsiprymnodontidae | h (l), b (u) | b (u), nt (l) | h? | b | 38 |
|  | Potoroidae | h (l), b (u) | b (u), nt (l) | h? | b | 38 |
|  | Macropodidae | h (l), b (u) | nt | b | b | 38 |
| Eulipotyphla | Erinaceidae | b | b | b | b | 38 |
|  | Nesophontidae | - | - | - | - | 38 |
|  | Solenodontidae | h | b | b | b | 38 |
|  | Soricidae | b | b | b | b | 38 |
|  | Talpidae | b | b | b | b | 38 |
| Pholidota | Manidae | nt | nt | nt | nt | 38 |
| Carnivora  Carnivora | Felidae | b | h | b | b | 38 |
|  | Viverridae | b | h | b | b | 38 |
|  | Eupleridae | b | h | b | b | 38 |
|  | Nandiniidae | b | h | b | b | 38 |
|  | Herpestidae | b | h | b | b | 38 |
|  | Hyaenidae | b | h | b | b | 38 |
|  | Canidae | b | h | b | b | 38 |
|  | Ursidae | b | h | b | b | 38 |
|  | Otariidae | b | h | b (undif.) | b (undif.) | 38 |
|  | Odobenidae | b | hl (u), b (l) | b | nt | 38 |
|  | Phocidae | b | h | b | b | 38 |
|  | Mustelidae | b | h | b | b | 38 |
|  | Mephitidae | b | h | b | b | 38 |
|  | Procyonidae | b | h | b | b | 38 |
|  | Ailuridae | b | h | b | b | 38 |
| Chiroptera | Pteropodidae | b | h | b | b | 38 |
|  | Rhinolophidae | b | h | h | b | 38 |
|  | Hipposoderidae | b | h | h | b | 38 |
|  | Megadermatidae | b | h | b | b | 37 |
|  | Rhinopomatidae | b | h | b | b | 38 |
|  | Craseonycteridae | b | h | h | b | 38 |
|  | Emballonuridae | b | h | b | b | 38 |
|  | Nycteridae | b | h | b | b | 38 |
|  | Myzopodidae | b | h | h | b | 38 |
|  | Mystacinidae | b | h | h | b | 38 |
|  | Phyllostomidae | b | h | h? | b | 38 |
|  | Mormoopidae | b | h | b | b | 38 |
|  | Noctilionidae | b | h | b | b | 38 |
|  | Furipteridae | b | h? | h? | b | 38 |
|  | Thyropteridae | b | h | b | b | 38 |
|  | Natalidae | b | h | b | b | 38 |
|  | Molossidae | h (u), b (l) | h | h | b | 38 |
|  | Vespertilionidae | b | h | b | b | 38 |
| Perissodactyla | Equidea | h | nt | h | h | 38 |
|  | Tapiridae | b | b | b | b | 38 |
|  | Rhinocerotidae | nt | nt | h | h | 38 |
| Artiodactyla | Suidae | b | hl | b | b | 38 |
|  | Tayassuidae | b | hl | b | b | 38 |
|  | Hyppopotamidae | hl | hl | b | h? | 38 |
|  | Camelidae | h | b | h | h | 38 |
|  | Tragulidae | b (l), nt (u) | hl (u), nt (l) | b | b | 38 |
|  | Moschidae | b (l), nt (u) | hl (u), nt (l) | b | b | 38 |
|  | Giraffidae | b (l), nt (u) | b (l), nt (u) | b | b | 38 |
|  | Cervidae | b (l), nt (u) | b (l), nt (u), hl (u) | b | b,h | 38 |
|  | Antilocapridae | b (l), nt (u) | b (l), nt (u) | h | h | 38 |
|  | Bovidae | nt | nt | h | h | 38 |
| Cetacea | Balenidae | p | p | p | p | 38 |
|  | Balaenopteridae | p | p | p | p | 38 |
|  | Eschrichtiidae | p | p | p | p | 38 |
|  | Neobalaenidae | p | p | p | p | 38 |
|  | Delphinidae | b (undif.) | b (undif.) | b (undif.) | b (undif.) | 38 |
|  | Monodontidae | hl, nt | b (undif.) | b (undif.) | b (undif.) | 38 |
|  | Phocoenidae | b (undif.) | b (undif.) | b (undif.) | b (undif.) | 38 |
|  | Physeteridae | b (l), nt (u) (undif.) | b (l), nt (u) (undif.) | b (l), nt (u) (undif.) | b (l), nt (u) (undif.) | 38 |
|  | Platanistidae | b (undif.) | b (undif.) | b (undif.) | b (undif.) | 38 |
|  | Iniidae | b (undif.) | b (undif.) | b (undif.) | b (undif.) | 38 |
|  | Ziphiidae | nt, b | nt, b | nt, hl | nt | 38 |
| Scandentia | Tupaiidae | h | b | b | b | 38 |
|  | Ptilocercidae | h | b | b | b | 38 |
| Lagomorpha | Ochotonidae | hl | nt | hl | hl | 38 |
|  | Prolagidae | - | - | - | - | 38 |
|  | Leporidae | hl | nt | nt | hl | 38 |
| Rodentia  Rodentia | Aplodontiidae | hl | nt | hl | hl | 38 |
|  | Sciuridae | hl | nt | nt | h | 38 |
|  | Gliridae | hl | nt | b | b | 38 |
|  | Castoridae | hl | nt | nt | hl | 38 |
|  | Heteromyidae | hl | nt | b,h,hl | b,h,hl | 38 |
|  | Geomyidae | hl | nt | hl | hl | 38 |
|  | Dipodidae | hl | nt | b (u), nt (l) | b | 38 |
|  | Platacanthomyidae | hl | nt | nt | h | 38 |
|  | Spalacidae | hl | nt | nt | h | 38 |
|  | Calomyscidae | hl | nt | nt | b | 38 |
|  | Nesomyidae | hl | nt | nt | b,h | 38 |
|  | Cricetidae | hl | nt | nt | b,hl | 38 |
|  | Muridae | hl | nt | nt | b | 38 |
|  | Anomaluridae | hl | nt | b | b | 38 |
|  | Pedetidae | hl | nt | hl | hl | 38 |
|  | Ctenodactylidae | hl | nt | (lost early in life) | hl | 38 |
|  | Diatomyidae | hl | nt | h | h | 38 |
|  | Bathyergidae | hl | nt | h | h | 38 |
|  | Hystricidae | hl | nt | h | h | 38 |
|  | Petromuridae | hl | nt | h | h | 38 |
|  | Thryonomyidae | hl | nt | h | h | 38 |
|  | Eretizontidae | hl | nt | b | b | 38 |
|  | Chinchillidae | hl | nt | hl | hl | 38 |
|  | Dinomyidae | hl | nt | h, hl? | h, hl? | 38 |
|  | Caviidae | hl | nt | nt | hl | 38 |
|  | Dasyproctidae | hl | nt | h | h | 38 |
|  | Cuniculidae | hl | nt | h | h | 38 |
|  | Ctenomyidae | hl | nt | h | h | 38 |
|  | Octodontidae | hl | nt | h | h | 38 |
|  | Abrocomidae | hl | nt | hl | hl | 38 |
|  | Echimyidae | hl | nt | b | b | 38 |
|  | Myocastoridae | hl | nt | h | h | 38 |
|  | Heptaxodontidae | - | - | - | - | 38 |
| Dermoptera | Cynocephalidae | b | b | b | b | 38 |
| Primates | Cheirogaleidae | h (l), b (u) | h | b | b | 38 |
|  | Lemuridae | h | b | b | b | 38 |
|  | Lepilemuridae | h (l), nt (u) | h | b | b | 38 |
|  | Indriidae | h (l), b (u) | h | b | b | 38 |
|  | Daubentoniidae | hl | nt | b | b | 38 |
|  | Lorisidae | h (l), b (u) | h | b | b | 38 |
|  | Galagidae | h (l), b (u) | h | b | b | 38 |
|  | Tarsiidae | h | b | b | b | 38 |
|  | Cebidae | b | h | b | b | 38 |
|  | Aotidae | b | h | b | b | 38 |
|  | Pitheciidae | h | h | b | b | 38 |
|  | Atelidae | b | h | b | b | 38 |
|  | Cercopithecidae | h? | h | b | b | 38 |
|  | Hylobatidae | b | h | b | b | 37 |
|  | Hominidae | b | b | b | b | 38 |
| Xenartha | Dasypodidae | nt | nt | h | h | 38 |
|  | Bradypodidae | nt | nt | h | h | 38 |
|  | Megalonychidae | nt | nt | nt | nt | 38 |
|  | Cyclopedidae | nt | nt | nt | nt | 38 |
|  | Myrmecophagidae | nt | nt | nt | nt | 38 |
| Tubulidentata | Orycteropodidae | nt | nt | h | h | 38 |
| Afrosoricida | Tenrecidae | b | b | b | b | 38 |
|  | Chrysochloridae | b | b | b | b | 38 |
| Macroscelidea | Macroscelididae | b | b | b | b | 38; 37 |
| Sirenia | Dugongidae | hl (u), nt (l), p | nt, p | h | h, hl | 38 |
|  | Trichechidae | nt | nt | b | b | 38 |
| Proboscidea | Elephantidae | hl | nt | h | h | 38 |
| Hyracoidea | Procaviidae | hl | nt | h | h | 38 |

**Table S2: Tooth types description of mammalian taxa in Figure 2**. Grey shading = Fossil taxa; White shading = Extent taxa. Dent. = Dentine; Ena. = Enamel; nb = number; (-) = no data available.

| **Infraclass** | **Order** | **Family** | ***Species*** | **Time**  **(Ma)** | **Node nb** | **Tooth types** | | | | **Tooth crown** | **Sources** |
| --- | --- | --- | --- | --- | --- | --- | --- | --- | --- | --- | --- |
|  |  |  |  |  |  | **Incisor** | **Canines** | **Premolars** | **Molars** |  |  |
| - | Morganucodonta | Morganucodontidae | *Morganucodon oehleri* | 196,5 |  | Brachydont | Brachydont | Brachydont | Brachydont | Enamel | 38 |
| - |  | Morganucodontidae | *Morganucodon watsoni* | 196,5 |  | Brachydont | Brachydont | Brachydont | Brachydont | Enamel | 38 |
| - | Docodonta | Docodontidae | *Haldanadon exspectatus* | 155,5 |  | Brachydont | Brachydont | Brachydont | Brachydont | Enamel | 38 |
| - | Australosphenida | Steropodontidae | *Steropodon galmani* | 112,0 | 1 | - | - | - | Brachydont | Enamel | 38 |
| MONOTREMATA | Monotremata | Tachyglossidae | *Megalibgwilia robusta* | 23,53 | 2 | No teeth | No teeth | No teeth | No teeth | - | 17 |
|  |  | Ornithorynchidae | *Obdurodon insignis* | 28,4 | 3 | No teeth | No teeth | No teeth | Brachydont | Enamel? | 17 |
|  |  | Tachyglossidae | *Tachyglossus aculeatus* | Recent |  | No teeth | No teeth | No teeth | No teeth | - | 38 |
|  |  | Ornithorynchidae | *Ornithorhyncus anatinus* | Recent |  | No teeth | No teeth | No teeth | Brachydont? | Keratinous | 38 |
| - | Dryolestida | Paurodontidae | *Henkelotherium guimarotae* | 155,5 | 4 | ? | Brachydont | Brachydont | Brachydont | Enamel | 17 |
| - | *incertae sedis* | Spalacotheriidae | *Zhangheotherium quinquecuspidens* | 130,0 | 5 | Brachydont | Brachydont | Brachydont | Brachydont | Enamel | 17 |
| - |  | *incertae sedis* | *Eomaia scansoria* | 130,0 |  | Brachydont | Hypsodont | Brachydont | Brachydont | Enamel | 38 |
| MARSUPIALIA | Deltatheroida | - | *Sinodelphys szalayi* | 130,0 | 6 | Brachydont | Hypsodont | Brachydont | Brachydont | Enamel | 38 |
|  |  | Microbiotheriidae | *Khasia cordillerensis* | 64,5 | 7 | - | - | - | - | - | - |
|  | Didelphimorphia | Peradectidae | *Peradectes minor* | 65,0 | 8 | Brachydont | Hypsodont | Brachydont | Brachydont | Enamel | 38 |
|  |  | - | *Pucadelphys andinus* | 64,5 |  | Brachydont | Hypsodont | Brachydont | Brachydont | Enamel | 38 |
|  |  | Microbiotheriidae | *Dromiciops gliroides* | Recent |  | Brachydont | Hypsodont | Brachydont | Brachydont | Enamel | 38 |
|  |  | Didelphidae | *Didelphis virgiana* | Recent |  | Brachydont | Hypsodont | Brachydont | Brachydont | Enamel | 38 |
| - | Cimolesta | Cimolestidae | *Maelestes gobiensis* | 83,5 |  | Brachydont | Hypsodont | Brachydont | Brachydont | Enamel | 22 |
| - | - | Zalambdalestidae | *Zalambdalestes lechei* | 83,5 |  | lower hypsodont? | Brachydont | Brachydont | Brachydont | Enamel | 17 |
| - | - | Asioryctidae | *Ukhaatherium nessovi* | 83,5 |  | Brachydont | Hypsodont | Brachydont | Brachydont | Enamel | 17 |
| PLACENTALIA | - | - | *Riostegotherium sp.* | 59,0 | 9 | - | - | - | - | - | 26 |
|  | Pilosa  Pilosa | - | *Protamandua rothi* | 17,5 | 10 | No teeth | No teeth | No teeth | No teeth | - | 26 |
|  |  | - | *Pseudoglyptodon chilensis* | 36,0 | 11 | - | - | Hypselodont | Hypselodont | Dentine | 26 |
|  |  | - | *Hapalops elongatus* | 17,5 |  | No teeth | Hypselodont | Hypselodont | Hypselodont | Dentine | 22 |
|  |  | Myrmecophagidae | *Tamandua tetractyla* | Recent |  | No teeth | No teeth | No teeth | No teeth | - | 38 |
|  | Cingulata | Dasypodidae | *Dasypus novemcinctus* | Recent |  | No teeth | No teeth | Hypsodont | Hypsodont | Dentine | 38 |
|  | Macroscelidea | Macroscelidae | *Chambius kassarinensis* | 55,8 | 12 | - | - | Brachydont | Brachydont | Enamel | 26 |
|  | Leptictida | Leptictidae | *Leptictis dakotensis* | 35,0 |  | Brachydont | Brachydont | Brachydont | Brachydont | Enamel | 22 |
|  | Leptictida | Leptictidae | *Prodiacodon crustulum* | 65,0 | 13 | - | Brachydont | Brachydont | Brachydont | Enamel | 21 |
|  | Macroscelidea | Macroscelidae | *Rhynchocyon cirnei* | Recent |  | No teeth | Hypsodont | Brachydont | Hypsodont | Enamel | 38 |
|  | Afrosoricida | - | *Protenrec tricuspis* | 23,3 | 14 | - | Brachydont | Brachydont | Brachydont | Enamel | 22 |
|  |  | - | *Eochrysochloris tribosfenus* | 33,9 | 15 | - | - | - | - | - | - |
|  |  | Tenrecidae | *Echinops telfairi* | Recent |  | Brachydont | Brachydont | Brachydont | Brachydont | Enamel | 38 |
|  |  | Chrysochloridae | *Amblysomus hottentotus* | Recent |  | Brachydont | Brachydont | Brachydont | Brachydont | Enamel | 38 |
|  | Tubulidentata | Orycteropodidae | *Myorycteropus africanus* | 13,65 | 16 | No teeth | No teeth | Hypselodont | Hypselodont | Dentine | 20 |
|  |  | Orycteropodidae | *Orycteropus afer* | Recent |  | No teeth | No teeth | Hypselodont | Hypselodont | Dentine | 38 |
|  | Hyracoidea | Pliohyracidae | *Titanohyrax tantalus* | 55,8 | 17 | - | - | Brachydont? | Brachydont? | Enamel | 26 |
|  |  | Procaviidae | *Procavia capensis* | Recent |  | Hypselodont | No teeth | Hypsodont | Hypsodont | Enamel | 38 |
|  | Paenungulata | - | *Simpsonotus praecursor* | 62,5 | 18 | - | - | - | - | - | - |
|  | Notoungulata | Isotemnidae | *Thomashuxleya externa* | 54,0 |  | Brachydont | Hypsodont | Hypsodont? | Hypsodont? | Enamel | 22 |
|  | Xenungulata | Carodniidae | *Carodnia vieirai* | 59,0 |  | Hypsodont | Hypsodont | Brachydont | Brachydont | Enamel | 22 |
|  | Proboscidea | - | Eritherium azzouzorum | 58,7 | 19 | Hypsodont | - | Brachydont | Brachydont | Enamel | 9 |
|  |  | - | *Barytherium grave* | 37,2 | 20 | Hypsodont | Brachydont | Brachydont | Brachydont | Enamel | 26 |
|  |  | - | *Loxodonta africana* | Recent |  | Hypselodont | No teeth | Hypsodont | Hypsodont | Dent./Ena. | 38 |
|  |  | - | *Moerithrium lyonsi* | 33,9 |  | Hypsodont | Brachydont | Brachydont | Brachydont | Enamel | 26 |
|  | Sirenia | - | *Prorastomus sirenoides* | 55,8 | 21 | - | - | - | - | - | - |
|  |  | Trichechidea | *Trichechus manatus* | Recent |  | No teeth | No teeth | Brachydont | Brachydont | Enamel | 38 |
|  | Scandentia  Scandentia | Tupaiidae | *Eodendrogale parvum* | 48,6 | 22 | - | - | Brachydont | Brachydont | Enamel | 26 |
|  |  | Tupaiidae | *Tupaia glis* | Recent |  | Hypsodont | Brachydont | Brachydont | Brachydont | Enamel | 38 |
|  |  | Tupaiidae | *Ptilocercinae gen. et sp. nov.* | 9,0 | 23 | Hypsodont? | Brachydont? | Brachydont? | Brachydont? | Enamel | 26 |
|  |  | Ptiloceridae | *Ptilocercus lowii* | Recent |  | Hypsodont | Brachydont | Brachydont | Brachydont | Enamel | 38 |
|  | Dermoptera | Cynocephalidae | *Dermotherium major* | 37,0 | 24 | Brachydont | Brachydont | Brachydont | Brachydont | Enamel | 26 |
|  |  | Cynocephalidae | *Galeopterus variegatus* | Recent |  | Brachydont | Brachydont | Brachydont | Brachydont | Enamel | 38 |
|  |  | Cynocephalidae | *Cynocephalus volans* | Recent |  | Brachydont | Brachydont | Brachydont | Brachydont | Enamel | 38 |
|  | Primates | - | *Purgatorius coracis* | 65,0 | 25 | Brachydont | Brachydont | Brachydont | Brachydont | Enamel | 26 |
|  |  | Adapidae | *Cantius torresi* | 55,8 | 26 | Brachydont | Brachydont | Brachydont | Brachydont | Enamel | 26 |
|  |  | Notharctidae | *Notharctus tenebrosus* | 50,3 |  | Brachydont | Hypsodont | Brachydont | Brachydont | Enamel | 22 |
|  |  | - | *Karanisia clarki* | 39,0 | 27 | - | - | - | - | - | - |
|  |  | Lemuridae | *Lemur catta* | Recent |  | Hypsodont | Brachydont | Brachydont | Brachydont | Enamel | 38 |
|  |  | Omomyidae | *Teilhardina brandti* | 55,8 | 28 | Brachydont | Brachydont | Brachydont | Brachydont | Enamel | 26 |
|  |  | Omomyidae | *Tarsius eocaenus* | 45,0 | 29 | Hypsodont? | Brachydont | Brachydont | Brachydont | Enamel | 26 |
|  |  | Tarsiidae | *Tarsius syrichta* | Recent |  | Hypsodont | Brachydont | Brachydont | Brachydont | Enamel | 38 |
|  |  | *incertae sedis* | *Branisella boliviana* | 27,0 | 30 | Brachydont | Brachydont | Brachydont | Brachydont | Enamel | 36 |
|  |  | Cebidae | *Saimiri sciureus* | Recent |  | Brachydont | Hypsodont | Brachydont | Brachydont | Enamel | 38 |
|  |  | Oligopithecidae | *Catopithecus browni* | 35,0 | 31 | Brachydont | Hypsodont | Brachydont | Brachydont | Enamel | 30 |
|  |  | Hominidae | *Homo sapiens* | Recent |  | Brachydont | Brachydont | Brachydont | Brachydont | Enamel | 38 |
|  | - | Alagomyidae | *Tribosphenomys minutus* | 60,0 |  | Hypselodont | No teeth | Brachydont | Brachydont | Enamel | 22 |
|  | - | Eurymylidae | *Rhombomylus turpanensis* | 53,0 |  | Hypselodont | No teeth | Brachydont | Brachydont | Enamel | 19 |
|  | - | - | *Mimotona wana* | 65,5 | 32 | Hypselodont | No teeth | Brachydont | Brachydont | Enamel | 26 |
|  | - | - | *Gomphos elkema* | 56,0 |  | Hypselodont? | No teeth | Hypsodont | Hypsodont | Enamel | 14 |
|  | - | - | *Dawsonolagus antiquus* | 54,0 |  | Hypselodont | No teeth | - | Hypsodont | Enamel | 22 |
|  | Lagomorpha | Leporidae | *Leporidae indet.* | 53,0 | 33 | Hypselodont | No teeth | No teeth | Hypselodont | Enamel | 22 |
|  |  | Leporidae | *Oryctolagus cuniculus* | Recent |  | Hypselodont | No teeth | No teeth | Hypselodont | Enamel | 38 |
|  | Rodentia  Rodentia | Ischyromyidae | *Paramys delicatus* | 55,4 |  | Hypselodont | No teeth | Brachydont | Brachydont | Enamel | 22 |
|  |  | Chapattimyidae | *Cocomys lingchaensis* | 56,0 |  | Hypselodont | No teeth | Brachydont | Brachydont | Enamel | 22 |
|  |  | Sciuridae | *Oligospermophilus douglassi* | 38,0 | 34 | - | - | Brachydont | Brachydont | Enamel | 13 |
|  |  | Sciuridae | *Ictidomys tridecemlineatus* | Recent |  | Hypselodont | No teeth | No teeth | Hypsodont | Enamel | 38 |
|  |  | Castoridae | *Agnotocastor sp.* | 38,0 | 35 | Hypselodont | No teeth | Brachydont | Brachydont | Enamel | 2 |
|  |  | Castoridae | *Castor canadensis* | Recent |  | Hypselodont | No teeth | No teeth | Hypselodont | Enamel | 38 |
|  |  | Sciuravidae | *Sciuravus sp.* | 56,8 | 36 | Hypselodont? | No teeth | - | Brachydont | Enamel | 40 |
|  |  | - | *Nonomys simplicidens* | 46,2 | 37 | Hypselodont | No teeth | No teeth | Brachydont | Enamel | 3 |
|  |  | Muridae | *Rattus norvegicus* | Recent |  | Hypselodont | No teeth | No teeth | Brachydont | Enamel | 38 |
|  |  | Hystricidae | *Gaudeamus* |  | 38 | Hypselodont | No teeth | Hypsodont | Hypsodont | Enamel | 28 |
|  |  | Caviidae | *Cavia porcellus* | Recent |  | Hypselodont | No teeth | No teeth | Hypselodont | Enamel | 38 |
|  | Eulipotyphla | - | *Protungulatum donnae* | 65,0 | 39 | - | Brachydont | Brachydont | Brachydont | Enamel | 22 |
|  |  | Talpidae | *Eotalpa belgica* | 37,2 | 40 | - | - | Brachydont | Brachydont | Enamel | 32 |
|  |  | Talpidae | *Talpa europaea* | Recent |  | Brachydont | Brachydont | Brachydont | Brachydont | Enamel | 38 |
|  |  | Solenodontidae | *Solenodon paradoxus* | Recent |  | Hypsodont | Brachydont | Brachydont | Brachydont | Enamel | 38 |
|  |  | Soricidae | *Domnina gradata* | 46,2 | 41 | - | - | Brachydont | Brachydont | Enamel | 31 |
|  |  | Soricidae | *Sorex araneus* | Recent |  | Brachydont | Brachydont | Brachydont | Brachydont | Enamel | 38 |
|  |  | Erinaceidae | *Litolestes ignotus* | 61,7 | 42 | Brachydont | Brachydont | Brachydont | Brachydont | Enamel | 25 |
|  |  | Erinaceidae | *Erinaceus europaeus* | Recent |  | Brachydont | Brachydont | Brachydont | Brachydont | Enamel | 38 |
|  | Pholidota | Escavadodontidae | *Escavadodon zygus* | 63,3 | 43 | - | Brachydont | Brachydont | Brachydont | Enamel | 27 |
|  |  | Metacheiromyidae | *Metacheiromys marshi* | 50,3 |  | - | Hypsodont | Brachydont | Brachydont | Enamel | 26 |
|  |  | Manidae | *Euromanis krebsi* | 48,4 | 44 | No teeth | No teeth | No teeth | No teeth | - | 6 |
|  |  | Manidae | *Manis pentadactyla* | Recent |  | No teeth | No teeth | No teeth | No teeth | - | 38 |
|  | Creodonta | Hyaenodontidae | *Prolimnocyon atavus* | 56,8 | 45 | No teeth? | Hypsodont? | Brachydont | Brachydont | Enamel | 7 |
|  |  | Hyaenodontidae | *Sinopa rapax* | 50,3 |  | Brachydont | Hypselodont? | Brachydont | Brachydont | Enamel | - |
|  | Carnivoramorpha | Viverravidae | *Protictis haydenianus* | 56,8 | 46 | Brachydont | Hypsodont | Brachydont | Brachydont | Enamel | 4, 11 |
|  |  | Miacidae | *Oodectes herpestoides* | 55,0 | 47 | Brachydont | Hypsodont | Brachydont | Brachydont | Enamel | 26 |
|  |  | Miacidae | *Vulpavus profectus* | 50,3 |  | Brachydont | Hypsodont | Brachydont | Brachydont | Enamel | 26 |
|  |  | Miacidae | *Vulpavus ovatus* | 50,3 |  | Brachydont | Hypsodont | Brachydont | Brachydont | Enamel | 26 |
|  | Carnivora | Canidae | *Hesperocyon gregarius* | 46,2 | 48 | Brachydont | Hypsodont | Brachydont | Brachydont | Enamel | 11 |
|  |  | Canidae | *Canis lupus* | Recent |  | Brachydont | Hypsodont | Brachydont | Brachydont | Enamel | 38 |
|  |  | - | *Stenoplesictis sp.* | 33,9 | 49 | - | Hypsodont | Brachydont | Brachydont | Enamel | 23 |
|  |  | Felidae | *Felis silvestris* | Recent |  | Brachydont | Hypsodont | Brachydont | Brachydont | Enamel | 38 |
|  | Chiroptera  Chiroptera | Pteropodidae | Pteropodidae indert. | 34,0 | 50 | Brachydont | Hypsodont | Brachydont | Brachydont | Enamel | 38 |
|  |  | Pteropodidae | *Pteropus giganteus* | Recent |  | Brachydont | Hypsodont | Brachydont | Brachydont | Enamel | 38 |
|  |  | Onychonycteridae | *Onychonycteris finneyi* | 52,5 |  | Brachydont | Hypsodont | Brachydont | Brachydont | Enamel | 29 |
|  |  | Icaronycteridae | *Icaronycterix index* | 52,5 |  | Brachydont | Hypsodont | Brachydont | Brachydont | Enamel | 12 |
|  |  | Archaeonycteridae | *Archaeonycteris praecursor* | 55,5 | 51 | Brachydont | Hypsodont? | - | Brachydont | Enamel | 12, 35 |
|  |  | Rhinopomatidae | *Rhinopoma sp.* | 9,0 | 52 | Brachydont | Hypsodont | Brachydont | Brachydont | Enamel | 22 |
|  |  | Rhinopomatidae | *Rhinopoma hardwickii* | Recent |  | Brachydont | Hypsodont | Brachydont | Brachydont | Enamel | 38 |
|  |  | Mormoopidae | *Pteronotus parnellii* | Recent | 53 | Brachydont | Hypsodont | Brachydont | Brachydont | Enamel | 22 |
|  |  | Mormoopidae | *Pteronotus parnellii* | Recent |  | Brachydont | Hypsodont | Brachydont | Brachydont | Enamel | 38 |
|  |  | - | *Wallia scalopidens* | 46,2 | 54 | - | - | - | - | - | - |
|  |  | Vespertilionidae | *Myotis lucifugus* | Recent |  | Brachydont | Hypsodont | Brachydont | Brachydont | Enamel | 38 |
|  |  | - | *Chibanycteris herbeti* | 31,5 | 55 | - | - | - | - | - | - |
|  |  | Nycteridae | *Nycteris thebaica* | Recent |  | Brachydont | Hypsodont | Brachydont | Brachydont | Enamel | 38 |
|  |  | Emballonuridae | *Tachypteron franzeni* | 47,0 | 56 | Brachydont | Hypsodont | Brachydont | Brachydont | Enamel | 33 |
|  |  | Emballonuridae | *Saccopteryx bilineata* | Recent |  | Brachydont | Hypsodont | Brachydont | Brachydont | Enamel | 38 |
|  | Condylarthra | Hyopsodontidae | *Hyopsodus paulus* | 56,8 | 57 | Brachydont | Brachydont | Brachydont | Brachydont | Enamal | 22 |
|  |  | Arctocyonidae | *Protungulatum donnae* | 65,0 |  | - | - | Brachydont | Brachydont | Enamel | 1 |
|  |  | Hyopsodontidae | *Litomylus dissentaneus* | 63,3 | 58 | - | - | - | - | - | - |
|  |  | Apheliscidae | *Apheliscus insidiosus* | 55,8 |  | - | - | Brachydont | Brachydont | Enamel | 22 |
|  |  | Phenacodontidae | *Tetraclaenodon puercensis* | 63,3 | 59 | - | - | Brachydont | Brachydont | Enamel | 22, 39 |
|  |  | Phenacodontidae | *Phenacodus intermedius* | 56,8 |  | - | Hypsodont | Brachydont | Brachydont | Enamel | 22 |
|  |  | Didolodontidae | *Escribania chubutensis* | 62,5 | 60 | - | - | - | Brachydont | Enamel | 8 |
|  |  | Didolodontidae | *Didolodus multicuspis* | 54,0 |  | - | - | Brachydont | Brachydont | Enamel | 22 |
|  |  | Protolipternidae | *Protolipterna ellipsodontoides* | 59,0 |  | - | - | Brachydont | Brachydont | Enamel | 22 |
|  | Artiodactyla | Mesonychidae | *Dissacus zanabazari* | 63,3 | 61 | - | Hypsodont | Brachydont | Brachydont | Enamel | 22 |
|  |  | Mesonychidae | *Mesonyx obtusidens* | 50,3 |  | - | Hypsodont | Brachydont | Brachydont | Enamel | 22 |
|  |  | Protocetidae | *Rodhocetus balochistanensis* | 47,0 |  | Brachydont | Brachydont | Brachydont | Brachydont | Enamel | 10 |
|  | Perissodactyla  Perissodactyla | Equidae | *Hyracotherium angustidens* | 55,4 | 62 | Brachydont | Hypsodont | Brachydont | Brachydont | Enamel | 18 |
|  |  | Equidae | *Mesohippus bairdi* | 42,0 |  | Brachydont | No teeth | Brachydont | Brachydont | Enamel | 22 |
|  |  | Equidae | *Miohippus grandis* | 38,0 | 63 | - | - | Brachydont | Brachydont | Enamel | 34 |
|  |  | Equidae | *Equus caballus* | Recent |  | Hypsodont | No teeth | Hypsodont | Hypsodont | Enamel | 38 |
|  | Artiodactyla | Cainotheriidae | *Cainotherium sp.* | 55,8 | 64 | Brachydont | Brachydont | Brachydont | Brachydont | Enamel | 24 |
|  |  | Camelidae | *Lama glama* | Recent |  | Hypsodont | Brachydont | Hypsodont | Hypsodont | Enamel | 38 |
|  |  | Cebochoeridae | *Cebochoerus minor* | 34,0 | 65 | Brachydont | Hypsodont | Hypsodont | Brachydont | Enamel | 24 |
|  |  | Suidae | *Sus scrofa* | Recent |  | Brachydont | Hypselodont | Brachydont | Brachydont | Enamel | 38 |
|  |  | Anthracotheriidae | *Elomeryx crispus* | 35,2 | 66 | - | No teeth | Brachydont | Brachydont | Enamel | 15 |
|  |  | Bovidae | *Bos taurus* | Recent |  | No teeth | No teeth | Hypsodont | Hypsodont | Enamel | 38 |
|  |  | Entelodontidae | *Archaeotherium mortoni* | 38,0 |  | Brachydont | Hypsodont | Brachydont | Brachydont | Enamel | 22 |
|  |  | Anthracotheriidae | *Merycopotamus sp.* | 13,9 | 67 | - | Hyspodont? | Brachydont | Brachydont | Enamel | 15 |
|  |  | Hippopotamidae | *Hippopotamus amphibius* | Recent |  | Hypselodont | Hypselodont | Brachydont | Hyspodont? | Enamel | 38 |
|  |  | Protocetidae | *Artiocetus clavis* | 47,0 |  | Brachydont | Brachydont | Brachydont | Brachydont | Enamel | 10 |
|  |  | Basilosauridae | *Basilosaurus cetoides* | 40,4 |  | Brachydont | Brachydont | Brachydont | Brachydont | Enamel | 22 |
|  |  | Eomysticetidae | *Eomysticetus carolinensis* | 27,0 | 68 | - | - | - | - | - | - |
|  |  | Neobalaenidae | *Caperea marginata* | Recent |  | plates | plates | plates | plates | Keratinous | 38 |
|  |  | - | *Simocetus rayi* | 32,0 | 69 | - | Brachydont | Brachydont | Brachydont | Enamel | 5 |
|  |  | Delphinidae | *Tursiops truncatus* | Recent |  | Brachydont (undif.) |  |  |  | Enamel | 38 |

**Table S3: Evolution of the different tooth categories – brachydont, hypsodont, hypselodont and no teeth**. The number of species that showed either of the tooth categories was counted based on the time range of the main eras. Then the percentages of species for each time period were calculated. The asterisk (*) shows the Era for which the sample is very low (less than 15 species). Ma = Million years.

|  |  |  | **Number of species** | | | | **% of species** | | | |
| --- | --- | --- | --- | --- | --- | --- | --- | --- | --- | --- |
| Era | Ma | Tooth type | **Incisor** | **Canine** | **Premolars** | **Molars** | **Incisor** | **Canine** | **Premolars** | **Molars** |
| Pliocene-Recent | **0-5** | *Brachydont* | 22 | 15 | 30 | 30 | 48,89 | 32,61 | 65,22 | 65,22 |
|  |  | *Hypsodont* | 7 | 13 | 6 | 9 | 15,56 | 28,26 | 13,04 | 19,57 |
|  |  | *Hypselodont* | 8 | 2 | 1 | 4 | 17,78 | 4,35 | 2,17 | 8,70 |
|  |  | *No teeth* | 8 | 16 | 9 | 3 | 17,78 | 34,78 | 19,57 | 6,52 |
| Miocene* | **5-28** | *Brachydont* | 2 | 3 | 5 | 5 | 28,57 | 33,33 | 55,56 | 55,56 |
|  |  | *Hypsodont* | 1 | 2 | 0 | 0 | 14,29 | 22,22 | 0,00 | 0,00 |
|  |  | *Hypselodont* | 0 | 1 | 2 | 2 | 0,00 | 11,11 | 22,22 | 22,22 |
|  |  | *No teeth* | 4 | 3 | 2 | 2 | 57,14 | 33,33 | 22,22 | 22,22 |
| Oligocene* | **28-38** | *Brachydont* | 5 | 5 | 10 | 12 | 71,43 | 45,45 | 76,92 | 92,31 |
|  |  | *Hypsodont* | 2 | 4 | 1 | 0 | 28,57 | 36,36 | 7,69 | 0,00 |
|  |  | *Hypselodont* | 0 | 0 | 1 | 1 | 0,00 | 0,00 | 7,69 | 7,69 |
|  |  | *No teeth* | 0 | 2 | 1 | 0 | 0,00 | 18,18 | 7,69 | 0,00 |
| Eocene | **38-55** | *Brachydont* | 14 | 4 | 22 | 24 | 66,67 | 17,39 | 84,62 | 85,71 |
|  |  | *Hypsodont* | 1 | 11 | 1 | 2 | 4,76 | 47,83 | 3,85 | 7,14 |
|  |  | *Hypselodont* | 5 | 1 | 0 | 1 | 23,81 | 4,35 | 0,00 | 3,57 |
|  |  | *No teeth* | 1 | 7 | 3 | 1 | 4,76 | 30,43 | 11,54 | 3,57 |
| Paleocene | **55-65** | *Brachydont* | 10 | 6 | 23 | 26 | 55,56 | 31,58 | 95,83 | 96,30 |
|  |  | *Hypsodont* | 2 | 8 | 1 | 1 | 11,11 | 42,11 | 4,17 | 3,70 |
|  |  | *Hypselodont* | 5 | 0 | 0 | 0 | 27,78 | 0,00 | 0,00 | 0,00 |
|  |  | *No teeth* | 1 | 5 | 0 | 0 | 5,56 | 26,32 | 0,00 | 0,00 |
| Cretaceous | **65-145** | *Brachydont* | 7 | 5 | 12 | 13 | 77,78 | 41,67 | 100,00 | 100,00 |
|  |  | *Hypsodont* | 1 | 6 | 0 | 0 | 11,11 | 50,00 | 0,00 | 0,00 |
|  |  | *Hypselodont* | 1 | 0 | 0 | 0 | 11,11 | 0,00 | 0,00 | 0,00 |
|  |  | *No teeth* | 0 | 1 | 0 | 0 | 0,00 | 8,33 | 0,00 | 0,00 |
| Jurassic* | **145-199** | *Brachydont* | 2 | 2 | 2 | 2 | 100,00 | 100,00 | 100,00 | 100,00 |
|  |  | *Hypsodont* | 0 | 0 | 0 | 0 | 0,00 | 0,00 | 0,00 | 0,00 |
|  |  | *Hypselodont* | 0 | 0 | 0 | 0 | 0,00 | 0,00 | 0,00 | 0,00 |
|  |  | *No teeth* | 0 | 0 | 0 | 0 | 0,00 | 0,00 | 0,00 | 0,00 |
| Triassic* | **199-250** | *Brachydont* | 2 | 2 | 2 | 2 | 100,00 | 100,00 | 100,00 | 100,00 |
|  |  | *Hypsodont* | 0 | 0 | 0 | 0 | 0,00 | 0,00 | 0,00 | 0,00 |
|  |  | *Hypselodont* | 0 | 0 | 0 | 0 | 0,00 | 0,00 | 0,00 | 0,00 |
|  |  | *No teeth* | 0 | 0 | 0 | 0 | 0,00 | 0,00 | 0,00 | 0,00 |

**Figure S1: Evolution of the tooth types in function of the tooth categories – incisors, canines, premolars and molars – throughout geological time**. The asterisks represent small sample size.

**
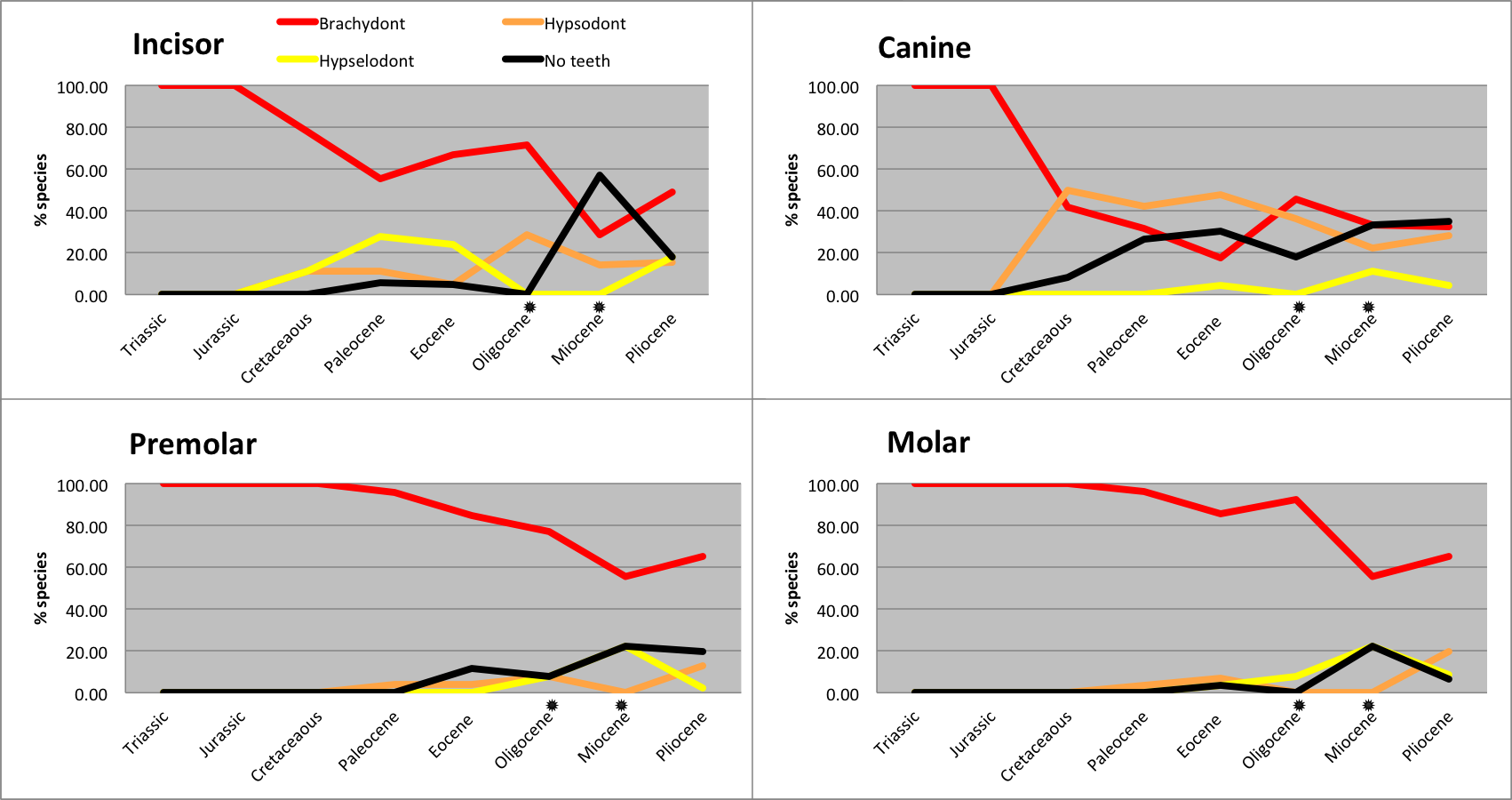
**

**References:**

(1) Archibald, J. D. (1982). A study of Mammalia and geology across the Cretaceous-Tertiary boundary in Garfield County, Montana. *University of California Publications in Geological Sciences*, **122**, 1-286.

(2) Emry, R. J. (1972). A new species of *Agnotocastor* (Rodentia, Castoridae) from the Early Oligocene of Wyoming. *American Museum Novitates*, **2485**, 1-7.

(3) Emry, R. J. (1981). New material of the Oligocene muroid rodent *Nonomys*, and its bearing on muroid origins. *American Museum Novitates*, **2712**, 1-14.

(4) Flynn, J. J. and Galiano, H. (1982). Phylogeny of Early Tertiary Carnivora, with a description of a new species of *Protictis* from the Middle Eocene of Northwestern Wyoming. *American Museum Novitates*, **2725**, 1-64.

(5) Fordyce, S. (2002). Simocetus rayi (Odontoceti: Simocetidae, New Family): A bizarre archaic Oligocene dolphin from the Eastern Noth Pacific. *Smithsonian Contributions to Paleobiology*, **93**, 185-222.

(6) Gaudin, T. J., Emry, R. J. and Wible, J. R. (2009). The Phylogeny of Living and Extinct Pangolins (Mammalia, Pholidota) and Associated Taxa: A Morphology Based Analysis. *Journal of Mammalian Evolution*, **16**, 235-305.

(7) Gebo, D. L. and Rose, K. D. (1993). Skeletal morphology and locomotor adaptation in *Prolimnocyon atavus*, an Early Eocene Hyaenodontid Creodont. *Journal of Vertebrate Paleontology*, **13**, 125-144.

(8) Gelfo, J. N., Ortiz-Jaureguizar, E. and Rougier, G. W. (2008). New remains and species of the ‘condylarth’ genus Escribania (Mammalia: Didolodontidae) from the Palaeocene of Patagonia, Argentina. *Earth and Environmental Science Transactions of the Royal Society of Edinburgh*, **98**, 127-138.

(9) Gheerbrant, E. (2009). Paleocene emergence of elephant relatives and the rapid radiation of African ungulates. *Proceedings of the National Academy of Science of America*, **106**, 10717-10721.

(10) Gingerich, P. D., Haq, M. u., Zalmount, I. S., Khan, I. H. and Malkani, S. M. (2001). Origin of whales from early Artiodactyls: hands and feet of Eocene Protocetidae from Pakistan. *Science*, **293**, 2239-2242.

(11) Gingerich, P. D. and Winkler, D. A. (1985). Systematics of Paleocoene Viverravidae in the bighorn basin ans clark's fork basin, Wyoming. *Contributions from the museum of paleontology: The University of Michigan*, **27**, 87-128.

(12) Gunnell, G. F. and Simmons, N. B. (2012). *Evolutionary history of bats: Fossils, molecules and morphology*, United Kingdom: Cambridge University PRess.

(13) Korth, W. W. (1987). Sciurid rodents form the Chardonian and Orellan (Oligocene) of Nebraska. *Journal of Paleontology*, **61**, 1247-1255.

(14) Kraatz, B. P., Meng, J., Weksler, M. and Li, C. (2010). Evolutionary patterns in the dentition of Duplicidentata (Mammalia) and novel trend in the molarization of premolars. *PloS One*, **5**, e12838.

(15) Lihoreau, F., Ducrocq, S., Antoine, P.-O., Vianey-Liaud, M., Rafaÿ, S.*, et al.* (2009). First complete skulls of *Elomeryx crispus* (Gervais, 1849) and ofProtaceratherium albigense (Roman, 1912) from a new Oligocene locality near Moissac (SW France). *Journal of Vertebrate Paleontology*, **29**, 242-253.

(16) Lim, W. K., Wang, K., Lefebvre, C. and Califano, A. (2007). Comparative analysis of microarray normalization procedures: effects on reverse engineering gene networks. *Bioinformatics (Oxford, England)*, **23**, i282-288.

(17) Luo, Z.-X., Kielan-Jaworowska, Z. and Cifelli, R. L. (2004). Evolution of dental replacement in mammals. *Carnegie Museum of Natural History*, **36**, 150-175.

(18) MacFadden, B. J. (1992). *Fossil horses: Systematics, paleobiology, and evolution of the family Equidae*, Cambridge, UK: Cambridge University Press.

(19) Meng, J., Hu, Y. and Li, C. (2003). The osteology of Rhombomylus (Mammalia, Glires): Implications for phylogeny and evolution of glires. *Bulletin of the American Museum of Natural History*, **275**, 1-247.

(20) Meredith, R. W., Gatesy, J., Murphy, W. J., Ryder, O. A. and Springer, M. S. (2009). Molecular decay of the tooth gene Enamelin (ENAM) mirrors the loss of enamel in the fossil record of placental mammals. *PLoS Genet*, **5**, e1000634.

(21) Novacek, M. (1977). A review of Paleocene and Eocene Leptictidae (Eutheria: Mammalia) from North America. *Paleo Bios*, **24**, 1-42.

(22) O'Leary, M. A., Bloch, J. I., Flynn, J. J., Gaudin, T. J., Giallombardo, A.*, et al.* (2013). The placental mammal ancestor and the post-K-Pg radiation of placentals. *Science*, **339**, 662-667.

(23) Peigné, S. and De Bonis, L. (1999). The genus Stenoplesictis Filhol (Mammalia, Carnivora) from the Oligocene deposits of the Phosphorites of Quercy, France. *Journal of Vertebrate Paleontology*, **19**, 566-575.

(24) Prothero, D. R. and Foss, S. E. (2007). *The evolution of Artiodactyls*, United States of America: The Johns Hopkins University Press.

(25) Rose, K. D. (2006). *The beginning of the age of mammals*, United States of America: The Johns Hopkins University Press.

(26) Rose, K. D. and Archibald, J. D. (2005). *The rise of placental mammals: origins and relationships of the major extant clades*, Unites States of America: The Johns Hopkins University Press.

(27) Rose, K. D. and Lucas, S. G. (2000). An early Paleocene palaeanodont (Mammalia, ?Pholidota) from New Mexico, and the origin of Palaeanodonta. *Journal of Vertebrate Paleontology*, **20**, 139-156.

(28) Sallam, H. M., Seiffert, E. R. and Simons, E. L. (2011). Craniodental morphology and systematics of a new family of Hystricognathous Rodents (Gaudeamuridae). *PloS One*, **6**, e16525.

(29) Simmons, N. B., Seymour, K. L., Habersetzer, J. and Gunnell, G. F. (2008). Primitive Early Eocene bat from Wyoming and the evolution of flight and echolocation. *Nature*, **451**, 818-821.

(30) Simons, E. L. and Rasmussen, D. T. (1996). Skull of *Catopithecus browni*, an Early Tertiary Catarrhine. *American Journal of Physical Anthropology*, **100**, 261-292.

(31) Simpson, G. G. (1941). A new oligocene insectivore. *American Museum Novitates*, **1150**, 1-3.

(32) Smith, R. (2007). Présence du genre Eotalpa (Mammalia, Talpidae) dans l'Oligocène inférieur de Belgique (Formation de Borgloon, MP 21). *Bulletin de l'Institut Royal des Sciences Naturelles de Belgique: Sciences de la Terre*, **77**, 159-165.

(33) Storch, G., Sigé, B. and Habersetzer, J. (2002). Tachypteron franzeni n.gen., n. sp., earliest emballonurid bat from the Middle Eocene of Messel (Mammalia, Chiroptera). *Paläontologische Zeitschrift*, **76**, 189-199.

(34) Strömberg, C. A. E. (2006). Evolution of hypsodonty in equids: testing a hypothesis of adaptation. *Paleobiology*, **32**, 236-258.

(35) Tabuce, R., Antunes, M. T. and Sigé, B. (2009). A new primitive bat from the earliest Eocene of Europe. *Journal of Vertebrate Paleontology*, **29**, 627-630.

(36) Takai, M., Anaya, F., Shigehara, N. and Setoghchi, T. (2000). New fossil materials of the earliest new world monkey, *Branisella boliviana*, and the problem of Platyrrhine origins. *American Journal of Physical Anthropology*, **111**, 263-281.

(37) Thenius, E. (1989). Zahne und Gebiss der Saugetiere. *Handbuch der Zoologie, v. 8, Mammalia*, ed. W. De Gruyter, New York.

(38) Ungar, P. S. (2010). *Mammal teeth: origin, evolution, and diversity*, United States of America: The John Hopkins University Press.

(39) West, R. W. (1970). Tetraclaenodon puercensis (Mammalia: Phenacodontidae), Golder formation, Paleocoene of California, and distribution of the genus. *Journal of Paleontology*, **44**, 851-857.

(40) Wood, A. E. (1949). Small mammals from the uppermost Eocene (Duchesnean) from Badwater, Wyoming. *Journal of Paleontology*, **23**, 556-565.
